# Alterations in cerebral resting state functional connectivity associated with social anxiety disorder and early life adversities

**DOI:** 10.1038/s41398-025-03301-x

**Published:** 2025-03-13

**Authors:** Melina Leypoldt, Ariane Wiegand, Matthias Munk, Sanja Drohm, Andreas J. Fallgatter, Vanessa Nieratschker, Benjamin Kreifelts

**Affiliations:** 1 Department of Psychiatry and Psychotherapy, Tuebingen Center for Mental Health (TüCMH), Tuebingen, Germany; 2 Max Planck Fellow Group for Precision Psychiatry, Max Planck Institute of Psychiatry, Munich, Germany; 3 German Center for Mental Health (DZPG), partner site Tuebingen, Tuebingen, Germany

**Keywords:** Psychiatric disorders, Neuroscience

## Abstract

Social Anxiety Disorder (SAD) involves fear of negative evaluation and social avoidance, impacting quality of life. Early life adversities (ELA) are recognized as risk factors for SAD. Previous research indicated inconsistent alterations in resting state functional connectivity (RSFC) in SAD, particularly in the prefrontal cortex and precuneus. This study investigated the interaction between SAD and ELA at the RSFC level. Functional magnetic resonance imaging (fMRI) was conducted on 120 participants (aged 19–48). Four groups were formed: low/ high ELA controls (n = 49, n = 22) and low/ high ELA SAD participants (n = 30, n = 19). Seed-based correlation analyses (SCA) and multi-voxel pattern analysis (MVPA) were applied. A network in which ELA moderates the neural correlates of SAD during the resting state was identified, involving key nodes like the subgenual anterior cingulate cortex, left middle frontal gyrus, and an area in the calcarine fissure/precuneus. Five distinct interaction patterns of SAD and ELA were observed, showcasing opposite RSFC patterns in individuals with SAD based on ELA experience. Results remained significant when controlled for general anxiety and depression measures. Emotional aspects of ELA played a significant role in these interactions. These findings stress the necessity of considering primarily emotional ELA as covariate in neuroimaging studies investigating SAD and potentially also other psychiatric disorders, addressing inconsistencies in prior research. The left middle frontal gyrus emerges as a link in the SAD-ELA interaction during resting state and anxiety-relevant stimulation. Longitudinal studies, starting from childhood, are needed to understand ELA’s impact on brain function and to identify potential neuromarkers for SAD predisposition post-ELA exposure.

## Introduction

Anxiety disorders, including social anxiety disorder (SAD), are among the most common mental illnesses, with a lifetime prevalence ranging from 9.2% to 28.7% [1]. SAD, a prevalent psychiatric condition [2], is marked by excessive fear in and avoidance of social situations [3], thus limiting quality of life [4]. Moreover, SAD is also associated with an increased risk of psychiatric comorbidity [5].

Negative life events, such as early life adversities (ELA), can contribute to SAD development [6]. ELA encompasses exposure during childhood or adolescence to circumstances requiring significant psychological, social, or neurobiological adaptation [7]. The overall global prevalence for different subtypes of ELA is estimated to be 12.7% for sexual abuse, 16.3% for physical neglect, 18.4% for emotional neglect, 22.6% for physical abuse and 36.3% for emotional abuse [8]. ELA have been identified as a frequent key contributory factor for many different psychiatric disorders in adulthood such as anxiety disorders [9], major depressive disorder (MDD) [10, 11] and post-traumatic stress disorder [12]. A history of ELA may influence nearly all clinical aspects of internalizing mental disorders including longer duration, greater risk of recurrence or relapse after a period of remission, increased comorbidities, and poorer treatment outcomes for psychotherapy as well as pharmacotherapy [13–15]. Also, ELA are clinically associated with an earlier onset of anxiety disorders [16] and depressive disorders [13] and greater symptom severity in current SAD [17, 18], as well as in MDD [13].

Extensive research has explored the neural correlates of SAD [19–21]. Resting state functional connectivity (RSFC) studies in individuals with SAD have yielded conflicting results, with both increased and decreased connectivity observed in various brain regions, including the amygdala [21], dorsolateral prefrontal cortex (dlPFC) [22, 23], anterior cingulate cortex (ACC) [23–25], and precuneus [22, 26].

ELA, on the other hand, are recognized as a common contextual factor in SAD [27], potentially leading to functional alterations and long-lasting behavioral effects, including changes in emotion processing [28], enhanced fear perception [29], and alterations in attentional control [28]. Investigations into the cerebral underpinnings of ELA are limited [30, 31]. RSFC studies on ELA have reported both increased and decreased connectivity of the amygdala with regions such as the medial prefrontal cortex [31–33], and decreased connectivity with the ACC [31] and precuneus [30], aligning with regions of altered RSFC reported in studies on SAD. The ACC and medial prefrontal cortex are thought to be involved in fear processing and the appraisal of negative emotions [34]. Threat experiences influence these brain circuits and increase the risk of psychopathology by impairing emotion regulation [35].

The interaction between SAD and ELA at the brain function level is largely unexplored, despite their evident clinical interconnections. Studies on the interaction between ELA and psychopathology in other internalizing disorders indicate altered RSFC in regions similar to those observed in SAD and ELA: In MDD moderating effects of ELA were detected in various brain regions including the occipital cortex [36–38], precuneus [39] and prefrontal regions [38–42]. Similarly, in panic disorder interactions with ELA were observed in occipital regions and in the fear network model including the ACC [43]. By contrast, in obsessive-compulsive disorder, ELA moderated RSFC in thalamus, prefrontal regions and insula indicating an influence on emotional processing [44].

As the first work in SAD focusing on the interaction with ELA at the neural level, Wiegand et al. investigated hemodynamic brain activation during the processing of fear-related stimuli based on a subsample from the present study. They observed increased activation in the left dlPFC and left medial superior frontal gyrus in individuals with SAD and low ELA but not in individuals with SAD and high ELA, suggesting that the cerebral response to anxiety-relevant stimuli is moderated by the experience of ELA [45]. However, no study has yet examined interactions between SAD and ELA on cerebral RSFC.

Given the role of ELA as a clinical moderator of SAD [14, 16–18] and previous suggestions of ELA as a confounding factor in neuroimaging studies on psychiatric disorders [46], the present study aimed to investigate how ELA experience might moderate RSFC in SAD. To the best of our knowledge, this is the first study focusing on the interaction of SAD and ELA in RSFC.

Interaction effects were examined in a stepwise approach, starting with a seed-based analysis informed by a review of the literature and including the results of a previous study on brain activation interactions of SAD and ELA during the processing of anxiety-relevant stimuli [45] performed in a subsample of the current study. We then extended the connectivity analyses using a hypothesis-free explorative approach employing multi-voxel pattern analysis (MVPA) and additionally tested whether the observed interaction effects of SAD and ELA on RSFC were linearly related to the clinical severity of ELA.

## Methods

### Participants

A total of 120 German-speaking individuals of European descent, aged 19 to 48, participated in the study in Tuebingen, Germany. Participants were diagnosed using the German version of the Structured Clinical Interview for DSM-IV (SCID) [47]. Of these, 49 fulfilled the diagnostic criteria for SAD. Social anxiety severity was measured using the Liebowitz Social Anxiety Scale (LSAS) [48], and a history of ELA was assessed using the Childhood Trauma Questionnaire (CTQ) [49, 50]. Participants were classified as having high levels of ELA if they exceeded the severity threshold in at least one of the five ELA categories of the CTQ, as proposed by Bernstein and Fink [49]: ≥15 for emotional neglect, ≥13 for emotional abuse, ≥10 for physical abuse or neglect, and ≥8 for sexual abuse. Based on these criteria, participants were divided into four groups: low ELA controls (Ce), high ELA controls (CE), low ELA SAD participants (Se), and high ELA SAD participants (SE). General anxiety and depressive symptoms were assessed using the State-Trait-Anxiety-Inventory (STAI) [51] and the Beck Depression Inventory (BDI II) [52]. The Mehrfachwahl-Wortschatz-Intelligenztest (MWT-B; [53]) was used to assess premorbid intelligence. See Table S1 for a detailed sample description. The reported study sample includes the sample reported by Wiegand et al. [45]. The study was performed with the approval of the University of Tuebingen ethics committee and in accordance with the ethical guidelines of the Declaration of Helsinki in the present amended version. Written informed consent was obtained from all participants before inclusion.

### Study procedure

Participants had two appointments within one week. The first appointment included demographic, clinical and psychometric assessments, while the second appointment involved a functional magnetic resonance imaging (fMRI) in resting state with eyes closed.

### Acquisition and preprocessing of fMRI data

The resting state fMRI scan using a 3 Tesla scanner (PRISMA, Siemens, Erlangen, Germany) took 10 min and comprised 405 volumes. Structural T1-weighted images were acquired (208 slices, repetition time (TR): 2400 ms, echo time (TE): 2.22 ms, voxel size: 0.8 × 0.8 × 0.8 mm^3^, field of view (FoV): 256 × 256 mm^2^, flip angle (FA): 8°). Functional images were obtained using multiband echo-planar imaging (EPI) sequences (72 slices, 2 mm thickness, TR: 1500 ms, TE: 34 ms, voxel size: 2x2x2mm^3^, FoV: 192 × 192 mm^2^, FA: 70°).

For preprocessing and statistical analyses SPM12 (Statistical Parametric Mapping, version 12; Wellcome Department of Cognitive Neurology, Institute of Neurology, University College London, UK; http://www.fil.ion.ucl.ac.uk/spm) and the CONN-toolbox (version 20b; https://www.nitrc.org/projects/conn) [54], implemented in SPM12 and based on MATLAB were used. Realignment of functional data and unwarping using a voxel displacement map derived from the field map were performed. Outlier scans were identified using ART [55] (framewise displacement > 0.9 mm or global blood oxygen level dependent (BOLD) signal changes > 5 SD [56, 57]). A reference BOLD image was computed for each participant by averaging all valid scans. Subsequently, all images were normalized into standard Montreal Neurological Institute (MNI) space [58], segmented into grey matter, white matter, and CSF [59] and smoothed with a Gaussian kernel (6 mm full width at half maximum).

### Data analysis

SPSS Statistics (Version 28.0.1.0; IBM) was used to analyze demographical and clinical data. The evaluation of the SAD × ELA interaction relied on an analysis of variance (ANOVA) with SAD and ELA as between-subject factors and gender and age as covariates of no interest.

The RSFC analyses were based on a general linear model (GLM). Denoising [60] included single-subject linear regression of confounding effects characterized by BOLD signal from white matter and CSF [61] (5 temporal components each [62]), motion regression [63] (12 components: 6 movement parameters and their first order derivatives), outlier scans [56] (here 63 factors), and effect of rest condition [54] (with first order derivative). Bandpass filtering [64] (0.008–0.09 Hz) was applied.

Individual seed-based connectivity maps were estimated and Fisher-transformed bivariate correlation coefficients from a weighted general linear model (weighted-GLM [65]) were calculated for each seed-target-pair.

At group-level, the interaction term as contrast of interest was defined as (Ce – CE) – (Se – SE) within the framework of an ANOVA. Significance was assessed two-tailed. In voxel-based analyses, cluster-level statistical thresholds were applied. We performed the following analyses:

1. Seed-based approaches [66]: a literature review on RSFC in SAD and ELA in PubMed (https://pubmed.ncbi.nlm.nih.gov) was conducted. Regions with RSFC alterations in both SAD and ELA and replicated for at least one of the factors were defined as regions of interest (ROIs). For a detailed description of this approach, see the Supplementary Material. 14 ROIs were determined: right and left amygdala, right and left hippocampus, right and left anterior cingulate, right angular gyrus, right supramarginal gyrus, right middle frontal gyrus and its orbital part, right superior frontal gyrus (medial part), left precuneus, left putamen and left middle temporal gyrus.
  1. A ROI-to-ROI analysis with the 14 ROIs was calculated [p < 0.05, uncorrected at connection level with false discovery rate (FDR) correction for the number of tested connections].
  2. Seed-based correlation analyses (SCA) with the 14 ROIs and two additional ROIs previously identified as showing SAD × ELA interactions during the processing of anxiety-relevant stimuli (left middle frontal gyrus; left medial superior frontal gyrus) in a subsample of the current study [45] were computed with a cluster-forming threshold of p < 0.001 (uncorrected) and a cluster threshold of p < 0.05, FDR-corrected. Cluster-level FDR-corrected results were further Bonferroni-corrected for the number of ROIs (p < 0.05/ 16 = 0.003125).
2. As a voxel-wise whole brain approach, an MVPA [67] (with four components) was performed. The fc-MVPA assesses the connectivity patterns between each voxel and the rest of the brain. Using principal component analysis, a low-dimensional multivariate representation of each voxel’s connectivity pattern with all other voxels is calculated [67]. The MVPA was performed with a height threshold of p < 0.001 (uncorrected) and p < 0.05 with FDR-correction at cluster level for multiple comparisons. Results were further explored with post-hoc SCA on significant MVPA clusters applying the same statistical thresholds. Convergence of the resulting RSFC patterns was tested using conjunction analyses with a minimum t-statistic [68] to identify common nodes functionally linking the MVPA clusters.

The Automated Anatomic Labelling Atlas (AAL) [69] was used to describe all significant clusters.

### Post-hoc tests

The mean Z-transformed correlation values from significant clusters were extracted, and t-tests were performed to further investigate the observed interaction effects. Bonferroni correction was applied to account for multiple comparisons for the number of tested connections (p < 0,05/36). Using multiple linear regression, we tested the stability of the results when controlling for measures of general anxiety and depression (STAI-X1/2 and BDI II). Additionally, we examined differences in the linear associations of RSFC with the severity of ELA between healthy individuals and those with SAD. The Pearson correlation between the mean Z-transformed RSFC correlation values and the total and subscale scores of the CTQ were calculated separately for participants with and without SAD and compared using the Fisher-Z-test. Results were Holm-Bonferroni corrected for multiple testing (total CTQ score with correction for 36 connections, subscores with correction for 36 connections and 5 subscores: 36*5 = 180).

## Results

### Sample data

The characteristics of the study sample are shown in Table 1.

**Table 1:** Sample characteristics.

	Control		Social anxiety disorder				
Characteristics	low early life adversity n = 49	high early life adversity n = 22	low early life adversity n = 30	high early life adversity n = 19	Effect of social anxiety disorder	Effect of early life adversity	Effect of social anxiety disorder × early life adversity
Age, yr	25.00 ± 3.77	27.77 ± 7.40	24.27 ± 6.01	27.58 ± 8.58	F = 0.16 p = 0.694	F = 6.72 p = 0.011	F = 0.05 p = 0.819
Sex, n	31 F, 18 M	12 F, 10 M	22 F, 8 M	13 F, 6 M	F = 1.63 p = 0.204	F = 0.53 p = 0.468	F = 0.04 p = 0.839
LSAS score	16.16 ± 19.39	22.86 ± 18.07	67.60 ± 24.86	74.68 ± 26.60	F = 146.59 p < 0.001	F = 2.61 p = 0.109	F = 0.002 p = 0.964
CTQ score	29.22 ± 4.37	46.73 ± 9.89	32.67 ± 5.49	56.26 ± 14.81	F = 16.51 p < 0.001	F = 165.56 p < 0.001	F = 3.64 p = 0.059
State–Trait Anxiety Inventory, state score	32.57 ± 6.63	34.18 ± 8.79	43.60 ± 11.85	48.16 ± 12.69	F = 44.62 p < 0.001	F = 2.71 p = 0.102	F = 0.62 p = 0.433
State–Trait Anxiety Inventory, trait score	31.90 ± 7.67	37.32 ± 12.38	51.07 ± 11.64	55.47 ± 9.79	F = 90.94 p < 0.001	F = 6.30 p = 0.013	F = 0.07 p = 0.796
BDI II score	2.33 ± 3.94	3.86 ± 4.77	12.27 ± 9.74	17.68 ± 9.42	F = 77.42 p < 0.001	F = 6.63 p = 0.011	F = 2.06 p = 0.153
IQ (MWT-B)	105.24 ± 12.80	108.86 ± 11.23	108.30 ± 12.17	112.21 ± 14.47	F = 1.69 p = 0.197	F = 2.33 p = 0.130	F = 0.004 p = 0.953

Values given as mean ± standard deviation.

*F* female, *M* male, *LSAS* liebowitz social anxiety scale, *CTQ* childhood trauma questionnaire, *BDI II* beck depression inventory II, *MWT-B* mehrfachwahl-wortschatz-intelligenztest.

### ROI-to-ROI analysis

No significant interaction of SAD and ELA on RSFC between ROIs was observed. For results at an uncorrected level, see Table S2.

### Seed-based correlation analysis (SCA)

**Fig. 1:**
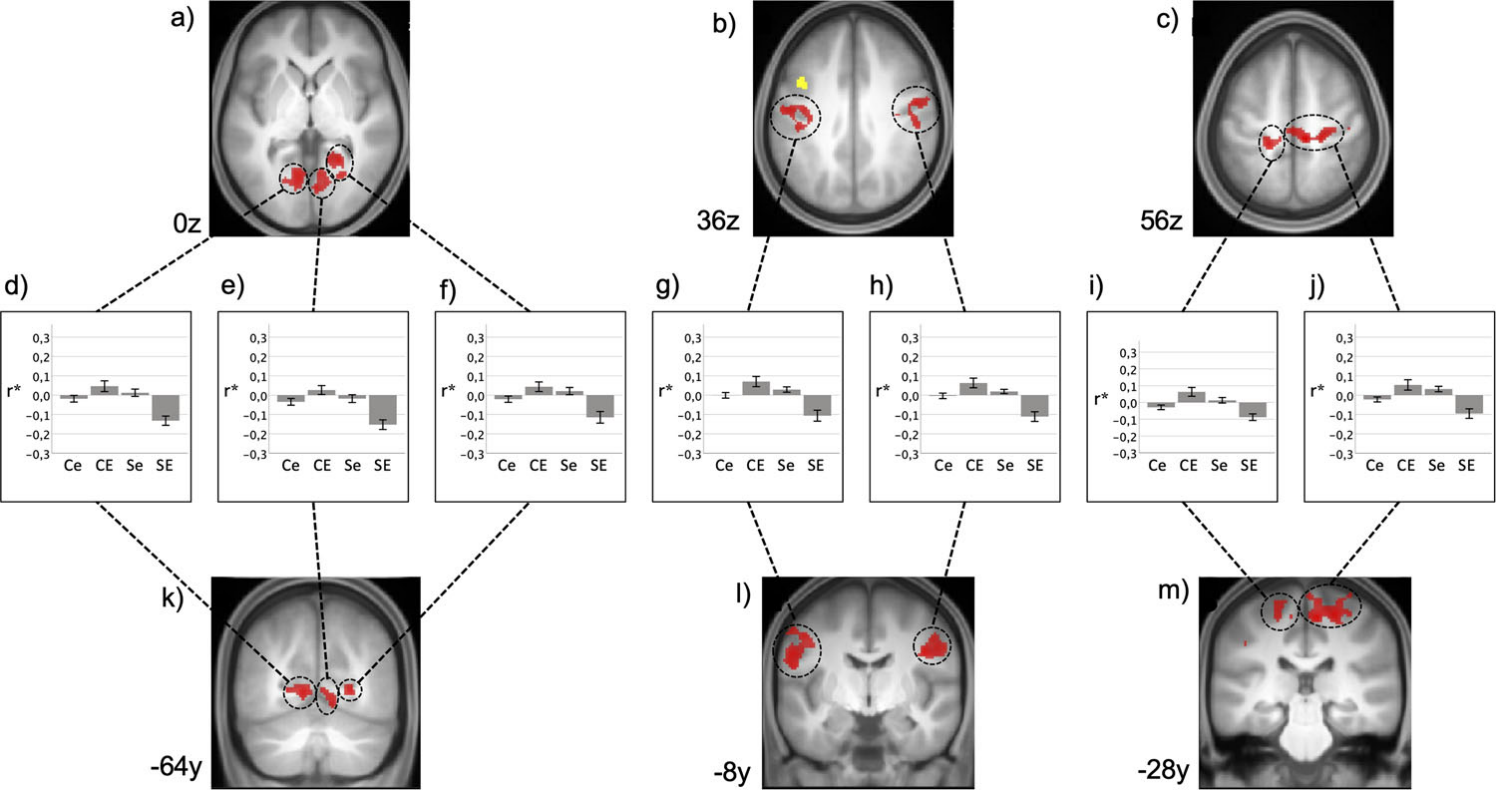
Resting state functional connectivity of left dlPFC. Interaction of SAD and ELA. Results are shown at p < 0.001, uncorrected at voxel-level and p < 0.05, FDR-corrected at cluster-level and additionally Bonferroni-corrected for 16 seeds projected onto the mean T1-weighted anatomical scan (n = 120, **a-c** transversal sectional images and **k-m** coronal sectional images). FDR = false discovery rate. Yellow: seed region in the dlPFC taken from Wiegand et al. [45]; Red: significant clusters of SCA (see Table S3 for details). **d**–**j** Diagrams depict the observed interaction patterns (Ce: controls with low ELA, CE: controls with high ELA, Se: participants with SAD and low ELA, SE: participants with SAD and high ELA). Z-transformed bivariate correlation coefficients (r*) are given on the y-axis. Error bars represent the standard error.

The dlPFC cluster with significant increased activation during the processing of fear-related stimuli [45] showed a significant interaction of SAD and ELA: we identified ten clusters located predominantly in the visual cortex, lateral and medial pre- and postcentral regions, and inferior parietal regions (Table S3; Fig. 1). Post-hoc analyses are reported in the context of observed spatial convergences with the MVPA results.

For the right hippocampus, we identified a cluster comprising the dorsolateral part of the right superior frontal gyrus and the right middle frontal gyrus (MNI: 30x, 66 y, 12z; 99 voxels) with an interaction effect, however not significant after Bonferroni correction. We did not observe any significant results for the other ROIs in the SCA. To test whether interactions between SAD and ELA regarding the amygdala had been missed due to its small size, we repeated the analyses with small volume correction [70] for the amygdala, however with no significant results.

### MVPA and post-hoc SCA

In the MVPA, we identified three significant clusters: left calcarine fissure/left precuneus (CAL, MNI: −26x, −60y, 12z, 70 voxels, p(FDR) = 0.002), subgenual anterior cingulate cortex (sACC, MNI: 6x, 10 y, −12z, 65 voxels, p(FDR) = 0.002), and left middle frontal gyrus (MFG, MNI: −34x, 8 y, 36z, 42 voxels, p(FDR) = 0.016).

**Fig. 2:**
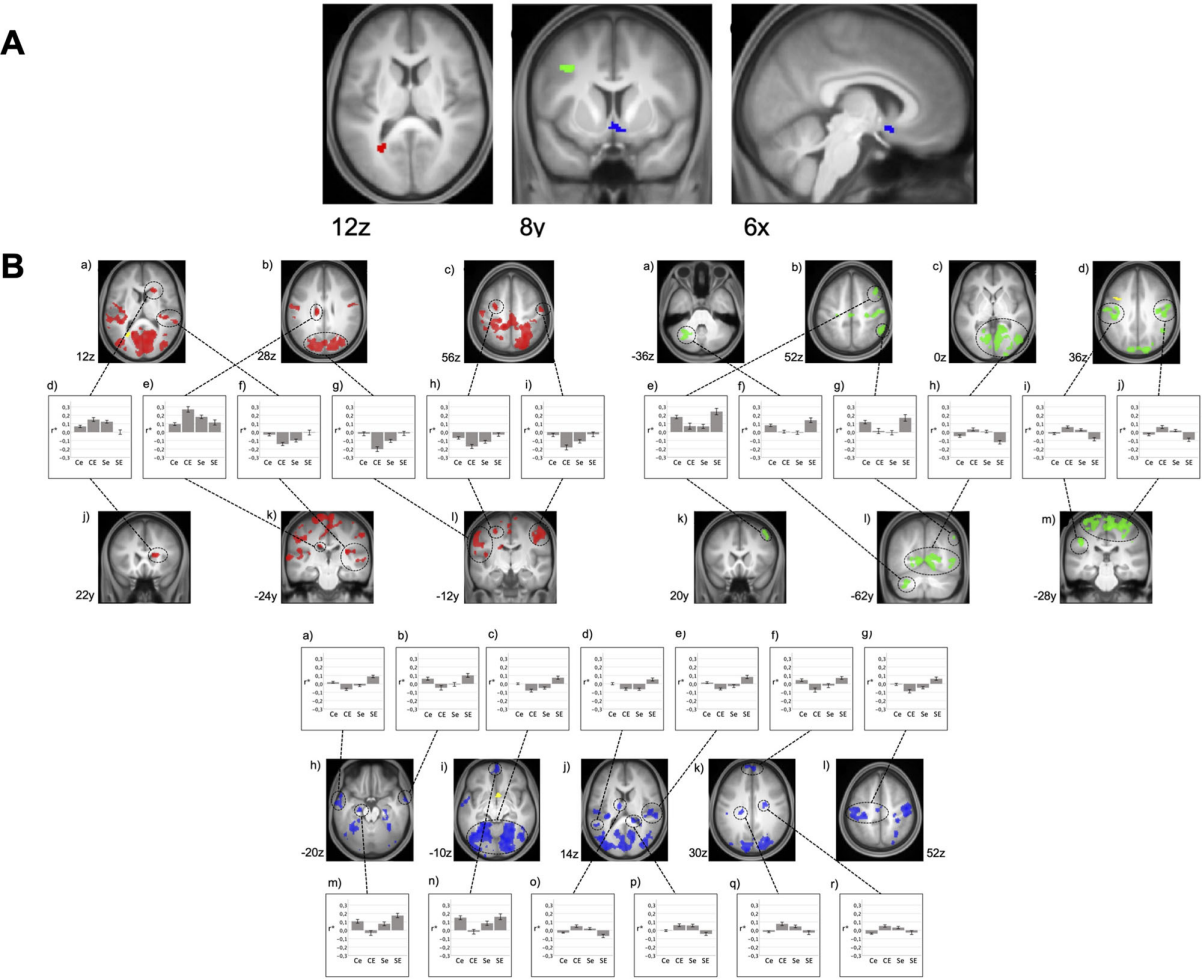
Multi voxel pattern analysis (MVPA) and post hoc explanatory seed-based correlation analysis (SCA). RSFC interactions between SAD and ELA. Results are shown at p < 0.001, uncorrected at voxel-level and p < 0.05, FDR-corrected at cluster-level projected onto the mean T1-weighted anatomical scan (n = 120); FDR = false discovery rate. **A** – MVPA: Red: left calcarine fissure/ precuneus (CAL); green: left middle frontal gyrus (MFG); blue: subgenual anterior cingulate cortex (sACC). **B** – SCA based on MVPA cluster seeds (yellow): Areas exhibiting an interaction of SAD and ELA with the left calcarine fissure/ precuneus (CAL, red, see Table S4 for details), with the left middle frontal gyrus (MFG, green, see Table S6 for details) and with the subgenual anterior cingulate cortex (sACC, blue, see Table S5 for details). Diagrams depict the observed interaction patterns (Ce: controls with low ELA, CE: controls with high ELA, Se: participants with SAD and low ELA, SE: participants with SAD and high ELA). Z-transformed bivariate correlation coefficients (r*) are given on the y-axis. Error bars represent standard error.

Post-hoc SCA of these clusters revealed a widespread set of cortical areas exhibiting significant interactions between SAD and ELA. Areas showing altered connectivity with the CAL are located in the visual cortex, medial and lateral pre- and post-central regions, right superior temporal gyrus, left superior and middle frontal gyrus, and bilateral caudate nucleus (Table S4, Fig. 2).

For the sACC, interactions of SAD and ELA are located in the visual cortex, in pre- and postcentral regions, in the middle and superior temporal gyrus, in the middle and superior frontal gyrus, in the left inferior parietal gyrus, as well as in the left hippocampus and thalamus and bilateral caudate nucleus (Table S5, Fig. 2).

The RSFC pattern for the MFG shows such interactions mainly in the visual cortex, pre- and postcentral regions, right middle frontal gyrus, inferior parietal regions and cerebellum (Table S6, Fig. 2).

### Functional Network

**Fig. 3:**
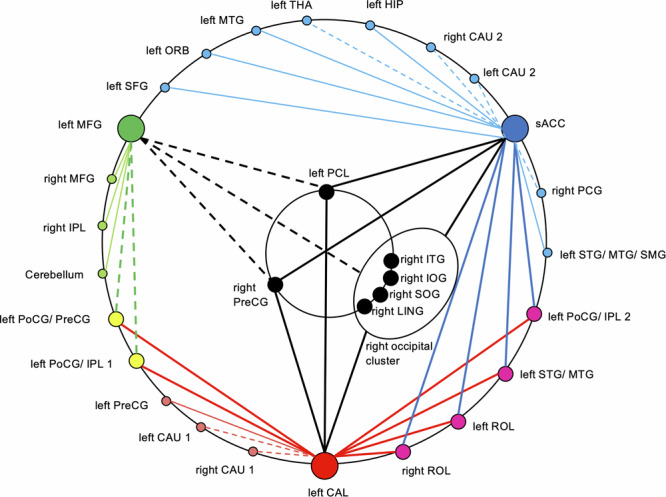
SAD × ELA-interaction functional network based on multivariate pattern analysis (MVPA) and post-hoc seed-based correlation analysis (SCA). MVPA: red: left calcarine fissure/precuneus (CAL), green: left middle frontal gyrus (MFG), blue: subgenual anterior cingulate cortex (sACC); SCA clusters connected to one MVPA cluster: light red, green, blue; SCA clusters connected to two MVPA clusters: yellow, magenta; SCA clusters connected to all three MVPA clusters: black. circled: four neighboring clusters treated as a functional unit (right occipital cluster) which came from the conjunction of the same SCA clusters. Continuous lines: positive interaction; broken lines: negative interaction. For details, see Table S4–7. Left CAU 1 = left caudate nucleus; left CAU 2 = left caudate nucleus; left HIP = left hippocampus; left ORB = left middle frontal gyrus, orbital part; left PCL = left paracentral lobule; left PoCG/ IPL 1 = left postcentral gyrus/ inferior parietal gyrus; left PoCG/ IPL 2 = left postcentral gyrus/inferior parietal gyrus; left PoCG/ PreCG = left postcentral gyrus,/ precentral gyrus; left PreCG = left precentral gyrus; left ROL = left Rolandic operculum; left SFG = left superior frontal gyrus; left STG/MTG = left superior temporal gyrus/ middle temporal gyrus; left STG/ MTG/ SMG = left superior temporal gyrus/ middle temporal gyrus/ supramarginal gyrus; left THA = left thalamus; right CAU 1 = right caudate nucleus; right CAU 2 = right caudate nucleus; right IOG = right inferior occipital gyrus; right IPL = right inferior parietal gyrus; right ITG = right inferior temporal gyrus; right LING = right lingual gyrus; right MFG = right middle frontal gyrus; right MTG = right middle temporal gyrus; right PCG = right posterior cingulate gyrus; right PreCG = right precentral gyrus; right ROL = right Rolandic operculum; right SOG = right superior occipital gyrus.

Conjunction analysis of the post-hoc SCAs of CAL, sACC, and MFG revealed spatial convergences between the RSFC patterns and identified a functional network consisting of 36 regions exhibiting interactions of SAD and ELA in their RSFC (Fig. 3, Table S4–S7). Six central clusters showing interactions in their RSFC with all three MVPA clusters were observed in the visual cortex, in pre- and postcentral regions, and in the middle and inferior temporal gyrus. Four of these came from three identical large SCA clusters and were thus treated as one functional unit in further analyses. Furthermore, six clusters exhibiting RSFC interactions with two of the three MVPA clusters were predominantly situated in the pre- and postcentral regions, in the superior and middle temporal gyrus, in left inferior parietal regions, and in the rolandic operculum (Table S7).

### Post-hoc analyses

#### Categorial interaction between SAD and ELA

**Fig. 4:**
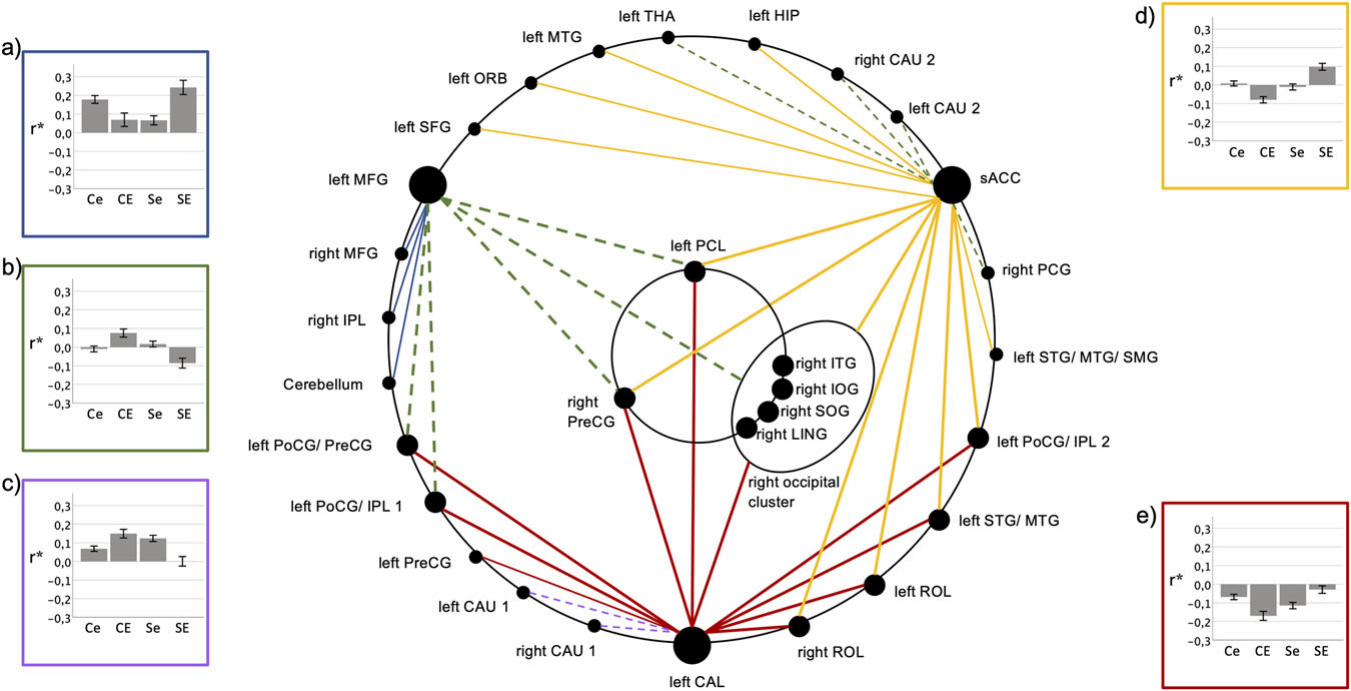
SAD × ELA-interaction types observed in the functional network. Connections between nodes are color-coded as indicated by the diagram frames. **a**–**e** Diagrams depict the observed interaction patterns (Ce: controls with low ELA, CE: controls with high ELA, Se: participants with SAD and low ELA, SE: participants with SAD and high ELA). Z-transformed bivariate correlation coefficients (r*) are given on the y-axis. Error bars represent standard error. Connections between (**a**) left and right MFG, (**b**) left MFG and left PoCG/ PreCG, (**c**) left CAL right CAU1, (**d**) sACC and left STG/ MTG, (**e**) left CAL and left PreCG exemplify the observed interaction patterns. For abbreviations of anatomical structures, see Fig. 3 and Table S4–7.

Five dominant interaction patterns of SAD and ELA, predominantly but not exclusively tied to the three central nodes of the network were identified. Uniformly, opposite ELA-related RSFC differences in SAD and controls were observed (Fig. 4, Table S8).

One pattern (Fig. 4, yellow, Table S8) observable in most of the sACC connections is characterized by a significantly stronger positive connectivity in SAD compared to healthy controls (HC) under the condition high ELA, whereas differences between the two groups were mostly lacking under the condition low ELA.

For the remaining connections of the sACC, the reverse pattern was found (Fig. 4, green, Table S8), with differences between HC and SAD in high ELA individuals and a predominant lack of significant differences between the low ELA subgroups. This pattern was also observed for the majority of the left MFG connections.

The third connectivity pattern was observed in most connections of CAL (Fig. 4, red, Table S8), characterized by a lower negative connectivity in participants with SAD and high ELA compared to high ELA controls, while the statistically non-significant converse difference is found under the low ELA condition. The fourth pattern found was the inversion of this pattern (Fig. 4, lilac, Table S8) in the remaining two connections of CAL.

A fifth pattern emerged for the remaining three connections of the MFG (Fig. 4, blue, Table S8), where SAD with high ELA is associated with stronger positive RSFC compared to high ELA HC. Conversely, for low ELA, individuals with SAD exhibit a lower positive correlation compared to HC.

**Fig. 5:**
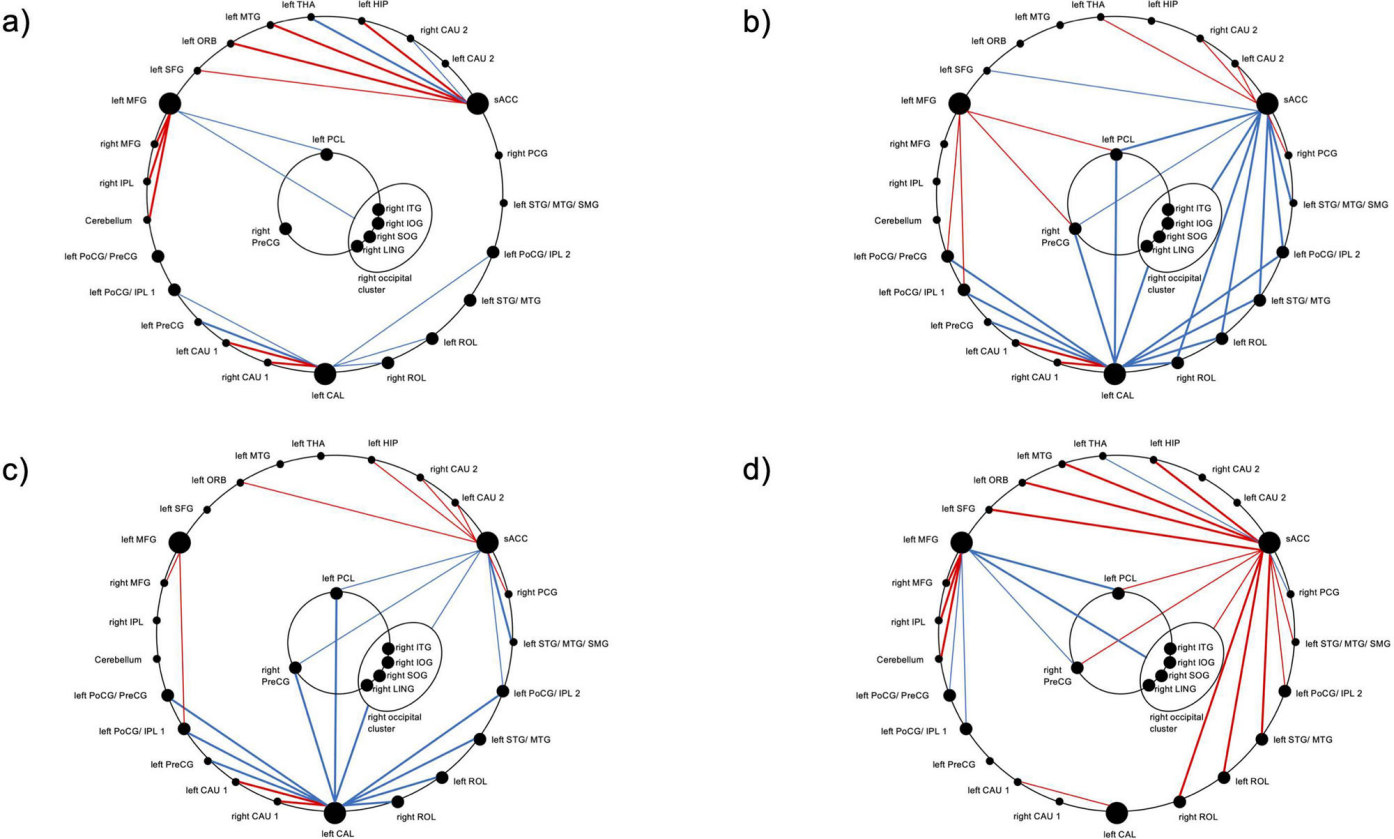
SAD × ELA-interactions. Group-wise functional states: (**a**) controls with low ELA, (**b**) controls with high ELA, (**c**) participants with SAD and low ELA, (**d**) participants with SAD and high ELA. Red: positive correlation; blue: negative correlation; thick lines: significance level of p < 0.001; thin lines: significance level of p < 0.05. For abbreviations of anatomical structures, see Fig. 3 and Table S4–7.

Next, the assessment of the absolute RSFC state of the network for each subject group (Fig. 5, Table S9) revealed a predominantly positive connectivity of the central node sACC and a predominantly negative connectivity of the central node CAL with other nodes in low ELA controls, as well as both negative and positive connectivity of the MFG. In contrast, in high ELA controls, a predominantly negative connectivity of the sACC and CAL and a positive connectivity of the MFG was found. Convergent with the results of the interaction analysis, a similar RSFC pattern was seen in participants with SAD and low ELA. By contrast, an increased positive connectivity (“hyperconnectivity”) of the sACC and almost complete lack of RSFC of the visual cortex (“disconnection”) were exhibited in participants with SAD and high ELA.

### Validation analyses

The interaction of SAD and ELA remained significant for all 36 connections when controlled for concomitant general anxiety (STAI-X1/2) and depressive symptoms (BDI II) (all F(1,116) ≥ 14.2, all p < 0.001).

### Parametric interaction between ELA severity and SAD

For all network connections, it was confirmed that the linear association between RSFC and ELA severity (i.e., CTQ scores) was significantly different between SAD and controls (Table S10, Fig. S2). Convergent with the results of the categorical analysis (Fig. 4), we observed a positive correlation of RSFC with ELA severity in SAD and a negative correlation in HC for most of the connections (Fig. 4, blue, yellow, and red connections). A reverse pattern was observed for the remaining connections (Fig. 4, green and lilac connections). Additional analyses with the CTQ subscores evidenced that the overall effect was best reflected in the subscales for emotional abuse (29/36 connections significant), emotional neglect (29/36 connections), and to a lesser degree physical neglect (21/36 connections), in contrast to the subscales for physical abuse (0/36 connections) and sexual abuse (1/36 connections).

### Convergence of literature-based SCA and MVPA-based network

A spatial convergence between the MFG node from the MVPA and the dlPFC cluster identified during the processing of anxiety-relevant stimuli by Wiegand et al. [45] was observed (Fig. S3A). Conjunction analysis of their SCA patterns identified nine significant spatial congruences (Table S11) which all correspond to nodes in the MVPA-based network: rLING, lPCL, rPreCG, lPoCG/PreCG, lPoCG/IPL1, lPoCG/lIPL2 (for abbreviations, see Table S7).

## Discussion

The primary discovery in this study highlights interactions between SAD and ELA manifesting in RSFC patterns of an extensive network. This network involves the sACC and visual/sensorimotor cortex regions, showing reversed RSFC correlates of SAD contingent on ELA experience. Notably, the left dlPFC, previously associated with similarly contrasting BOLD correlates of SAD during processing of disorder-relevant stimuli in a subsample of the current study [45], is implicated in this RSFC network. Our results align well with these prior findings [45], substantiating that neural correlates of SAD are influenced by ELA, not only during SAD-related stimulus confrontation but also during rest, and that the representations of these functional aspects are connected in the left dlPFC.

### RSFC network

We identified five distinct interaction patterns between SAD and ELA within the RSFC network. A consistent theme across these patterns was the opposite alteration of connectivity in SAD depending on the presence of ELA. The observation of the left MFG, CAL, and sACC as central nodes is consistent with findings from RSFC studies in SAD and ELA [21, 71]. Findings primarily in the prefrontal cortex have been contradictory with both increased and decreased RSFC in SAD [21–23] as well as ELA [31–33]. Moreover, both increased and decreased RSFC with the precuneus [22, 26] and ACC [23–25] in SAD have been reported. Here, our finding of interactions between SAD and ELA appears well suited to explain such discrepancies.

The central role of the occipital cluster in our RSFC network is marked by significant alterations in connectivity with the three main nodes partially aligning with RSFC alterations of the precuneus, sACC, and MFG with occipital regions [21], and increased activity during disorder-related stimulation in occipital regions, which has already been described in the context of SAD [20]. Convergently, moderating effects of ELA in occipital regions were repeatedly reported in other internalizing disorders, such as depression [36–38] or panic disorder [43]. Overall, the observed changes corroborate the notion of heightened attention to visual threats and increased activation of the visual system in SAD [72] as well as deficits in the perception of visual signals in individuals with a history of ELA and internalizing disorders [36]. In the context of MDD, this has been suggested as a neural correlate of how ELA may increase vulnerability to depression by influencing connectivity within specific occipital networks [37]. The sACC as part of the ventromedial prefrontal cortex plays a pivotal role in cognitive emotion processing and reappraisal [34] with a heightened involvement in the fear circuit in those with SAD [20].

Examining RSFC between the ACC and occipital regions has yielded inconsistent results, indicating both increased [25, 73] and decreased [74] connectivity in SAD. Decreased connectivity of the ACC with the inferior temporal gyrus [74] (which is part of this study’s right occipital cluster) in SAD compared to HC was also observed in our low ELA subsample, albeit at a level not surviving Bonferroni correction. In contrast, we observed previously described increased positive connectivity [25, 73] in high ELA SAD compared to high ELA controls (Table S8). These contradictory findings may be attributed to variations in ELA among study samples, highlighting ELA as a potential confounding factor in psychiatric neuroimaging studies. Our results align with previous findings suggesting ELA as a moderator of cerebral responses to anxiety-relevant stimuli [45], emphasizing the need to account for ELA in RSFC studies. Notably, RSFC correlates of SAD may be partially attributable to ELA or the interaction between ELA and SAD, further underscoring the necessity of considering ELA in future neuroimaging research [45].

Inconsistencies in RSFC findings between MFG and occipital regions [25, 74] might be attributable to the same methodological issues described above. Functionally, the MFG is implicated in attentional control and goal-oriented behavior [75, 76], with deficits observed in both SAD and individuals with ELA reflected in an attentional bias towards threat-related stimuli in SAD [77] and sustained attention to sad facial expressions in ELA [28]. Convergently, MFG and ACC are involved in fear, emotion processing, and emotional appraisal processes [34], while ELA are associated with impaired emotion processing [28] and hypersensitivity of fear-processing networks resulting in enhanced fear perception [29]. Functionally, such RSFC interactions between ELA and psychopathology in several prefrontal regions including MFG and ACC [41, 78] have been linked to resilience and susceptibility mechanisms focusing on emotion regulation in studies on MDD [78]. Altered connectivity of emotional and cognitive control regions have been suggested as a correlate of how ELA may lead to long-term neural changes in emotional responses, potentially increasing the vulnerability to MDD [38]. Analogously, a comparable mechanism may apply to SAD.

The third primary node, CAL, encompasses a portion of the precuneus and an adjacent cortex along the calcarine sulcus. Its functional discussion can be approached from either direction. The precuneus, linked to internal mental-state processes like self-referential processing and autobiographical memory retrieval [79], is part of the default mode network (DMN) [80, 81], along with other regions identified in our study’s network (e.g., posterior cingulate cortex, inferior parietal gyrus, middle temporal gyrus, and superior frontal gyrus) [82]. Our findings align with reports of both heightened and reduced RSFC of the precuneus in SAD [21, 22, 26] suggesting altered DMN function. Regarding the calcarine sulcus part, altered RSFC in this area in SAD [74, 82] is consistent with our observations, however not in individuals with ELA, potentially indicative of abnormal processing of visual information [74].

Interestingly, our results are in line with previous research demonstrating reversed RSFC differences between MDD and HC depending on a history of ELA [38, 39]. Also anatomically, these findings in MDD converge with the three key nodes of our network [39], as well as the visual cortex [38], and the inferior temporal gyrus (ITG) [39] as part of our occipital cluster. Moreover, decreased connectivity of the DMN (between the ITG and precuneus) was shown to mediate the relationship between ELA and depressive symptoms in MDD. It is suggested that individuals with MDD and ELA exhibit neural disruptions that result in negative biases for self-reference and affective information processing [39]. The similarity in connectivity patterns and involved structures may point to a similarity in the underlying pathological processes with biased self-reference and processing of emotional information as potential common elements in MDD and SAD.

Importantly, our findings remain significant after controlling for concomitant general anxiety and depressive symptoms, suggesting that social aspects of anxiety account for the observed interaction patterns.

From the perspective of group-wise RSFC states, it is striking that the connectivity patterns of HC with high ELA show similarities with individuals with SAD and low ELA, the source of which one can cautiously speculate upon. For one, SAD and the sequelae of ELA (i.e., adaptation processes with potential protective effects against symptom formation) might share certain neural resources. Alternatively, the connectivity pattern in SAD might reflect not only SAD symptomatology but also underlying adaption or compensation processes which do not effectively prevent SAD symptoms. Then again, the connectivity pattern in high ELA HC might be based on neuropsychological changes below the threshold of clinical SAD. Future studies are needed to clarify these hypotheses. Also, with respect to similar findings in MDD, adaptive mechanisms and their failure have been discussed as source of the reversed patterns. It is suggested that HCs with experience of ELA may develop altered connectivity in emotional processing networks as a compensatory response to ELA, which may prevent the development of depressive symptoms. In comparison, individuals with MDD and ELA show a reversed pattern indicating a decompensation process [38, 39].

Notably, the RSFC patterns show positive “hyperconnectivity” of the sACC in SAD with high ELA. In line with this, increased RSFC in the ACC could also be observed in MDD with ELA compared to MDD patients without ELA [42]. The observed “disconnection” of the visual cortex and precuneus in SAD with high ELA may speculatively be considered in the context of research on the interaction of SAD, ELA, and dissociative symptoms, suggesting that ELA moderate the association between social anxiety and dissociative symptoms [83]. The latter are characterized by emotional or physical detachment as a potential adaptation mechanism to cope with overwhelming emotional states following the experience of ELA [84]. The observed relative disconnection of the visual cortex in SAD with high ELA might be a correlate of an increased disposition to express dissociative symptoms. This fits in well with findings that clinically relevant dissociative symptoms (i.e., derealization and depersonalization disorder) are associated with decreased activity in the occipitotemporal cortex [85] and dorsal posterior cingulate [86], along with increased responses of frontal regions to aversive stimuli [86]. Also, the “disconnection” of the visual cortex in SAD with high ELA appears consistent with findings of a disconnection of the visual network in MDD with ELA [87]. Moreover, a decrease in cerebral blood flow in the visual cortex and a decrease in RSFC of the visual cortex in MDD with ELA has been discussed as indicating reduced sensory integration potentially linked to depressive symptoms [38].

### Parametric effects of ELA

The observation that the interaction of SAD and ELA correlates with the severity of ELA, which is best reflected by emotional abuse and emotional neglect, fits well with clinical findings that emotional maltreatment correlates with the severity of SAD [18], and emotional abuse was reported as the most common predictor of SAD [88]. Thus, our data highlight that emotional trauma in particular has a major influence on SAD psychopathology.

### Interaction of SAD and ELA: potential causes and research prospects

**Fig. 6:**
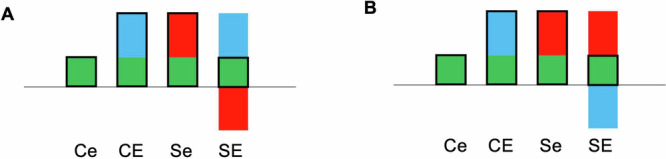
Hypothetical nature of the SAD × ELA interaction. **A** – Constant neural correlates of ELA and divergent neural correlates of SAD, e.g., (epi)genetically mediated. **B** – Interindividual variable correlates of ELA, e.g., genetically mediated and constant neural correlates of SAD. Green: functional connectivity unmoderated by ELA or SAD; blue: connectivity correlate of ELA; red: connectivity correlate of SAD; black outline: linear combination. Ce: controls with low ELA, CE: controls with high ELA, Se: participants with SAD and low ELA, SE: participants with SAD and high ELA.

Reductionistically and speculatively considering the RSFC interaction as a linear combination of SAD and ELA, we may consider the connectivity pattern in HC with low ELA to be healthy. HC with high ELA express a pattern combining healthy connectivity and the ELA correlate. Analogously, individuals with SAD and low ELA exhibit the sum of healthy connectivity and the SAD correlate. For those with SAD and high ELA, the connectivity pattern involves all three correlates, indicating an interaction term (Fig. 6). Two hypotheses arise: ELA could have an interindividually stable correlate with variability in the neurobiological correlate of SAD, depending on ELA (e.g. due to epigenetic effects [89, 90]). Or vice versa: invariable correlates underlie SAD, and the neurobiological sequelae of ELA differ interindividually and predictively between those who develop SAD and those who do not. Longitudinal studies in healthy children with high ELA and a control group would allow us to explore these hypotheses and potentially identify a neuromarker for SAD predisposition after ELA exposure.

## Limitations

While the CTQ is widely used in global research on ELA, it has limitations. Retrospective assessment may yield false negatives [91], and reported ELA levels may be influenced by subjective interpretation underlying cultural factors and societal influences [92]. Furthermore, there is no uniform construct of ELA [7] and further aspects like interpersonal loss, parental maladjustment, life-threatening physical illness, and family economic adversity are not sufficiently covered in the CTQ [27, 93]. Additionally, considering the relevance of timing and duration of exposure with the assumption of certain windows of vulnerability regarding the development of mental illness [94], the need for a more comprehensive and objective measure of ELA appears obvious. The inability to control for these various variables may limit the interpretability of our results. To address the issues concerning the impact of ELA on brain function in individuals with SAD but also other psychiatric disorders, longitudinal studies including comprehensive assessments of ELA starting during childhood and repeated at various timepoints up to adulthood are needed.

Regarding the lack of significant results in the ROI analysis, several factors should be discussed: Foremost, the use of anatomical structures as ROIs often poorly fitting the brain regions with observed connectivity changes may have considerably diminished the sensitivity of our ROI analyses. This strongly advocates the publication of masks indicating the exact location of observed effects as general standard. Moreover, beyond the influence of methodological differences between studies (e.g. connectivity metrics), the risk of false positive results in small-scale studies underlying ROI selection might have negatively influenced our analysis.

Further, our study is not perfectly matched with regard to gender and includes mainly younger participants. Research indicates gender-specific [95, 96] and age-related differences [97] in RSFC. Although we controlled for age and gender in our analyses, the observed effects may be more representative of female participants and a younger cohort. This should be considered when generalizing our findings to SAD patients across all genders and age groups.

Lastly, individuals with SAD have lower heart rate variability at rest [98], influencing the BOLD signal [99, 100]. Despite automatic correction of fMRI data for rhythmic signal fluctuations during preprocessing, differences between the four groups may still have influenced the results. Future studies should consider monitoring physiological parameters to better account for these factors during RSFC data preprocessing.

## Conclusion

The present study identifies an extensive functional network involving the sACC and visual and sensorimotor cortex regions in which RSFC changes in individuals with SAD are moderated by the experience of ELA. We identified the MFG as a structural link between the cerebral correlates of the interaction of SAD and ELA in resting state and during confrontation with anxiety-relevant stimuli [45]. Moreover, we provide first evidence for differences in the resting state neural underpinnings associated with SAD, depending on the experience of primarily emotional aspects of ELA, and we emphasize the necessity of taking ELA into account in resting state neuroimaging studies on SAD. Finally, our data highlight the need for longitudinal studies with onset in childhood to further explore the effects of ELA on brain function and possibly identify neuromarkers of a predisposition for SAD after exposure to ELA.

## Supplementary information

Supplementary Material

Supplementary Figure 1

Supplementary Figure 2

Supplementary Figure 3

## Data Availability

The neuroimaging data and SPM code underlying the findings reported in this study are available on reasonable scientific request from the corresponding author (benjamin.kreifelts@med.uni-tuebingen.de).
