# Supplementary material for: Alterations in cerebral resting state functional connectivity associated with social anxiety disorder and early life adversities
Source: Transl Psychiatry. 2025 Mar 13;15:80. doi: 10.1038/s41398-025-03301-x (PMC11906641; doi:10.1038/s41398-025-03301-x)
Supplement: Supplementary file 1 — Supplementary Material [file 41398_2025_3301_MOESM1_ESM.docx]

Supplementary Table S1. Clinical sample description for the four groups emerging from the factors SAD and ELA.

|  | **Ce** | **CE** | **Se** | **SE** |
| --- | --- | --- | --- | --- |
| Total participants | 49 | 22 | 30 | 19 |
| **Diagnosis** |  |  |  |  |
| Current depression (mild-moderate) | 0 | 0 | 2 | 3 |
| Dysthymia | 0 | 0 | 2 | 4 |
| Alcohol abuse | 1 | 1 | 0 | 2* |
| Panic disorder | 0 | 0 | 5 | 5 |
| Agoraphobia | 0 | 0 | 4 | 3 |
| Specific phobia | 4 | 0 | 6 | 4 |
| Obsessive-compulsive disorder | 0 | 0 | 0 | 2 |
| Generalized anxiety disorder | 0 | 0 | 3 | 2 |
| Post-traumatic stress disorder | 0 | 0 | 0 | 0 |
| **Medication** |  |  |  |  |
| Benzodiazepines | 0 | 0 | 2** | 0 |
| Antidepressants | 0 | 0 | 2*** | 3**** |

Ce: low ELA controls, CE: high ELA controls, Se: low ELA participants with SAD, SE: high ELA participants with SAD

*in the past

** infrequent use as needed

*** Escitalopram, Opipramol

**** Escitalopram, Citalopram, Fluoxetin

**Selection of regions of interest**

For the definition of the ROIs, we performed a review of the existing literature of RSFC in SAD and ELA in the Pubmed database (https://pubmed.ncbi.nlm.nih.gov) on 18.10.2021, whereby the following search terms were divided thematically into 3 blocks and combined with each other in different ways: Group 1: resting state fMRI, resting state, resting state functional connectivity; Group 2: social anxiety disorder, social phobia; Group 3: early life adversity, early life stress, childhood trauma, childhood maltreatment. This resulted in six search queries for SAD-rsfMRI studies, 12 search queries for ELA-rsfMRI studies and 24 search queries for SAD-ELA-rsfMRI studies. Only studies that described functional connectivity at rest using fMRI in adult subjects and performed a seed-based approach such as seed-based correlation analysis or ROI-to-ROI analysis and reported coordinates of observed clusters were included. Studies that measured functional connectivity after group therapy or pharmacotherapy or in children were not included. Our results were complemented by a meta-analysis by Mizzi et al. [1]. We translated the coordinates into AAL3 structures using the AAL3-Atlas (automated anatomical labelling atlas) [2] and selected the ROIs using a frequency display (see Supplementary Figure S1): regions were selected as ROIs if altered connectivity was reported in both SAD and ELA and replicated in at least one of the factors (i.e., number of studies >=2).

Supplementary Figure S1. Frequency display for the definition of the regions of interest.

The diagram describes the number of studies from the literature review regarding the localization of alterations in resting state functional connectivity associated with SAD and/or ELA. The number of studies is given on the x-axis while reported regions are displayed on the y-axis. *Brain structures included as regions of interest in the current study. a-t refer to the studies from the literature review.

a) Yuan M, Zhu H, Qiu C, et al. Group cognitive behavioral therapy modulates the resting-state functional connectivity of amygdala-related network in patients with generalized social anxiety disorder. BMC Psychiatry. 2016;16(1):198. doi:10.1186/s12888-016-0904-8

b) Dodhia S, Hosanagar A, Fitzgerald DA, et al. Modulation of Resting-State Amygdala-Frontal Functional Connectivity by Oxytocin in Generalized Social Anxiety Disorder. Neuropsychopharmacology. 2014;39(9):2061-2069. doi:10.1038/npp.2014.53

c) Prater KE, Hosanagar A, Klumpp H, Angstadt M, Luan Phan K. ABERRANT AMYGDALA-FRONTAL CORTEX CONNECTIVITY DURING PERCEPTION OF FEARFUL FACES AND AT REST IN GENERALIZED SOCIAL ANXIETY DISORDER. Depress Anxiety. 2013;30(3):234-241. doi:10.1002/da.22014

d) Jung YH, Shin JE, Lee YI, Jang JH, Jo HJ, Choi SH. Altered Amygdala Resting-State Functional Connectivity and Hemispheric Asymmetry in Patients With Social Anxiety Disorder. Front Psychiatry. 2018;9. doi:10.3389/fpsyt.2018.00164

e) Geiger MJ, Domschke K, Ipser J, et al. Altered executive control network resting-state connectivity in social anxiety disorder. The World Journal of Biological Psychiatry. 2016;17(1):47-57. doi:10.3109/15622975.2015.1083613

f) Hahn A, Stein P, Windischberger C, et al. Reduced resting-state functional connectivity between amygdala and orbitofrontal cortex in social anxiety disorder. Neuroimage. 2011;56(3):881-889. doi:10.1016/j.neuroimage.2011.02.064

g) van der Werff SJA, Pannekoek JN, Veer IM, et al. Resting-state functional connectivity in adults with childhood emotional maltreatment. Psychol Med. 2013;43(9):1825-1836. doi:10.1017/S0033291712002942

h) Rabany L, Diefenbach GJ, Bragdon LB, et al. Resting-State Functional Connectivity in Generalized Anxiety Disorder and Social Anxiety Disorder: Evidence for a Dimensional Approach. Brain Connect. 2017;7(5):289-298. doi:10.1089/brain.2017.0497

i) Pannekoek JN, Veer IM, van Tol MJ, et al. Resting-state functional connectivity abnormalities in limbic and salience networks in social anxiety disorder without comorbidity. European Neuropsychopharmacology. 2013;23(3):186-195. doi:10.1016/j.euroneuro.2012.04.018

j) Cui Q, Vanman EJ, Long Z, et al. Social anxiety disorder exhibit impaired networks involved in self and theory of mind processing. Soc Cogn Affect Neurosci. 2017;12(8):1284-1295. doi:10.1093/scan/nsx050

k) Manning J, Reynolds G, Saygin ZM, et al. Altered Resting-State Functional Connectivity of the Frontal-Striatal Reward System in Social Anxiety Disorder. PLoS One. 2015;10(4):e0125286. doi:10.1371/journal.pone.0125286

l) Hoffmann F, Viding E, Puetz VB, et al. Evidence for Depressogenic Spontaneous Thoughts and Altered Resting-State Connectivity in Adolescents With a Maltreatment History. J Am Acad Child Adolesc Psychiatry. 2018;57(9):687-695.e4. doi:10.1016/j.jaac.2018.05.020

m) Hakamata Y, Mizukami S, Izawa S, et al. Childhood trauma affects autobiographical memory deficits through basal cortisol and prefrontal-extrastriate functional connectivity. Psychoneuroendocrinology. 2021;127:105172. doi:10.1016/j.psyneuen.2021.105172

n) Minlan Y, Meng Y, Zhang Y, et al. Cerebellar Neural Circuits Involving Executive Control Network Predict Response to Group Cognitive Behavior Therapy in Social Anxiety Disorder. The Cerebellum. 2017;16(3):673-682. doi:10.1007/s12311-017-0845-x

o) Arnold Anteraper S, Triantafyllou C, Sawyer AT, Hofmann SG, Gabrieli JD, Whitfield-Gabrieli S. Hyper-Connectivity of Subcortical Resting-State Networks in Social Anxiety Disorder. Brain Connect. 2014;4(2):81-90. doi:10.1089/brain.2013.0180

p) Fadel E, Boeker H, Gaertner M, et al. Differential Alterations in Resting State Functional Connectivity Associated with Depressive Symptoms and Early Life Adversity. Brain Sci. 2021;11(5):591. doi:10.3390/brainsci11050591

q) Herringa RJ, Birn RM, Ruttle PL, et al. Childhood maltreatment is associated with altered fear circuitry and increased internalizing symptoms by late adolescence. Proceedings of the National Academy of Sciences. 2013;110(47):19119-19124. doi:10.1073/pnas.1310766110

r) Xu J, Guan X, Li H, Zhang M, Xu X. The Effect of Early Life Stress on Memory is Mediated by Anterior Hippocampal Network. Neuroscience. 2020;451:137-148. doi:10.1016/j.neuroscience.2020.10.018

s) Liao W, Xu Q, Mantini D, et al. Altered gray matter morphometry and resting-state functional and structural connectivity in social anxiety disorder. Brain Res. 2011;1388:167-177. doi:10.1016/j.brainres.2011.03.018

t) Philip NS, Sweet LH, Tyrka AR, Price LH, Bloom RF, Carpenter LL. Decreased default network connectivity is associated with early life stress in medication-free healthy adults. European Neuropsychopharmacology. 2013;23(1):24-32. doi:10.1016/j.euroneuro.2012.10.008

Supplementary Table S2. ROI-to-ROI-analysis

| **ROI 1** | **ROI 2** | **p-uncorrected** | **p(FDR)** |
| --- | --- | --- | --- |
| left middle temporal gyrus | left precuneus | p=0.020 | p=0.636 |
| right hippocampus | left precuneus | p=0.029 | p=0.636 |
| right superior frontal gyrus (medial part) | left hippocampus | p=0.038 | p=0.636 |
| right amygdala | left precuneus | p=0.040 | p=0.636 |
| left amygdala | left precuneus | p=0.041 | p=0.636 |
| right middle frontal gyrus | left hippocampus | p=0.042 | p=0.636 |

ROI= region of interest, FDR= false discovery rate

Supplementary Table S3. Resting state functional connectivity of the left dlPFC[3]: Interaction of SAD and ELA.

| **Anatomical region** | **Peak voxel MNI coordinates** | **Cluster size** | **p (FDR)** |
| --- | --- | --- | --- |
| left postcentral gyrus, left precentral gyrus, left inferior parietal but supramarginal and angular gyri | -56, -08, 28 | 485 | p<0.001* |
| right postcentral gyrus, right precentral gyrus, right supramarginal gyrus | 42, -10, 30 | 474 | p<0.001* |
| right precentral gyrus, right paracentral lobule, right postcentral gyrus, right supplementary motor area, left paracentral lobule | 10, -30, 56 | 436 | p<0.001* |
| left paracentral lobule, left postcentral gyrus, left precentral gyrus | -18, -32, 56 | 224 | p<0.001* |
| left lingual gyrus, left calcarine fissure and surrounding cortex | -10, -62, 02 | 213 | p<0.001* |
| right lingual gyrus, right calcarine fissure and surrounding cortex | 06, -76, -04 | 200 | p<0.001* |
| right lingual gyrus, right calcarine fissure and surrounding cortex | 26, -62, 04 | 166 | p=0.002* |
| left cuneus, left superior occipital gyrus | -08, -82, 28 | 127 | p=0.007 |
| right calcarine fissure and surrounding cortex, right inferior occipital gyrus, right middle occipital gyrus, right superior occipital gyrus | 16, -102, 02 | 85 | p=0.032 |
| left inferior temporal gyrus, left Temporal pole: middle temporal gyrus | -38, 00, -46 | 79 | p=0.037 |

dlPFC=dorsolateral prefrontal cortex from Wiegand et al. [3]; FDR= false discovery rate; voxel size 2x2x2mm^3^. * p<0.05 after Bonferroni correction for the number of seed regions (n=16).

Supplementary Table S4. Resting state functional connectivity of left calcarine fissure/ precuneus (CAL): Interaction of SAD and ELA.

| **Anatomical region** | **Peak voxel MNI coordinates** | **clustersize** | **p (FDR)** |
| --- | --- | --- | --- |
| right lingual gyrus, left calcarine fissure and surrounding cortex, left lingual gyrus, left postcentral gyrus, right calcarine fissure and surrounding cortex, left cuneus, right superior parietal gyrus, left middle occipital gyrus, right postcentral gyrus, right cuneus, left paracentral lobule, right middle occipital gyrus, left superior occipital gyrus, right superior occipital gyrus, right fusiform gyrus, right paracentral lobule, left precuneus, left fusiform gyrus, right precuneus | -42, -38, 8* | 23214 | p<0.001 |
| right precentral gyrus, right postcentral gyrus, right supramarginal gyrus | 46, -12, 42* | 901 | p<0.001 |
| right superior temporal gyrus, right rolandic operculum, right Heschl gyrus, right Insula | 32, -26, 16** | 460 | p<0.001 |
| left precentral gyrus, left superior frontal gyrus dorsolateral, left middle frontal gyrus **(abbr. lPreCG)** | -26, -10, 54 | 108 | p=0.026 |
| left caudate nucleus **(abbr. lCAU1)** | -12, -18, 28 | 120 | p=0.02 |
| right caudate nucleus **(abbr. rCAU1)** | 20, 24, 10 | 95 | p=0.037 |

FDR= false discovery rate, voxel size 2x2x2mm^3^. *Cluster overlaps with connectivity pattern of both subgenual anterior cingulate cortex (sACC) and left middle frontal gyrus (MFG) clusters from MVPA. ** Cluster overlaps with connectivity pattern of sACC cluster from MVPA. Only abbreviations for the clusters that do not significantly overlap with the connectivity patterns of the other MVPA clusters are listed. The remaining abbreviations can be found in Table S7 on the results of the conjunction analysis.

Supplementary Table S5. Resting state functional connectivity of the subgenual anterior cingulate cortex (sACC): Interaction of SAD and ELA.

| **Anatomical region** | **Peak voxel MNI coordinates** | **clustersize** | **p (FDR)** |
| --- | --- | --- | --- |
| right lingual gyrus, left middle occipital gyrus, left lingual gyrus, left calcarine fissure and surrounding cortex, right calcarine fissure and surrounding cortex, right middle occipital gyrus, left fusiform gyrus, right postcentral gyrus, right fusiform gyrus, left cuneus, right cuneus, right precentral gyrus, right superior occipital gyrus, left inferior occipital gyrus, left superior occipital gyrus, right middle temporal gyrus | 12, -68, -06* | 16294 | p<0.001 |
| left postcentral gyrus, left paracentral lobule, left precentral gyrus, left inferior parietal but supramarginal and angular gyri, left precuneus, left supplementary motor area, right paracentral lobule | -32, -32, 62* | 2368 | p<0.001 |
| left superior temporal gyrus, left middle temporal gyrus, left rolandic operculum, left Heschl gyrus, left Temporal pole: superior temporal gyrus, left inferior temporal gyrus, postcentral gyrus | -58, -22, 06** | 938 | p<0.001 |
| right superior temporal gyrus, right rolandic operculum, right Heschl gyrus, right middle temporal gyrus, right Insula | 48, -20, 18** | 465 | p<0.001 |
| left superior temporal gyrus, left middle temporal gyrus, left supramarginal gyrus **(abbr. lSTG/lMTG/lSMG)** | -50, -44, 10 | 185 | p<0.001 |
| left middle frontal gyrus orbital part, right middle frontal gyrus orbital part, left gyrus rectus **(abbr. lORB)** | 00, 64, -10 | 133 | p=0.004 |
| right middle temporal gyrus, right Temporal pole: middle temporal gyrus, right Temporal pole: superior temporal gyrus, right superior temporal gyrus **(abbr. rMTG)** | 64, 04, -20 | 124 | p=0.004 |
| left superior frontal gyrus medial, right superior frontal gyrus medial, left superior frontal gyrus dorsolateral **(abbr. lSFG)** | 02, 60, 32 | 113 | p=0.006 |
| left hippocampus, left parahippocampal gyrus **(abbr. lHIP)** | -20, -16, -22 | 65 | p=0.04 |
| left Thalamus, left caudate nucleus **(abbr. lTHA)** | -10, -08, 08 | 175 | p<0.001 |
| right posterior cingulate gyrus **(abbr. rPCG)** | 14, -38, 12 | 126 | p=0.004 |
| right caudate nucleus **(abbr. rCAU2)** | 24, -08, 30 | 91 | p=0.014 |
| left caudate nucleus **(abbr. lCAU2)** | -18, -18, 28 | 88 | p=0.015 |

FDR= false discovery rate, voxel size 2x2x2mm^3^. *Cluster overlaps with connectivity pattern of both left calcarine fissure/ precuneus (CAL) and left middle frontal gyrus (MFG) clusters of MVPA. ** Cluster overlaps with connectivity pattern of CAL cluster from MVPA. Only abbreviations for the clusters that do not significantly overlap with the connectivity patterns of the other MVPA clusters are listed. The remaining abbreviations can be found in Table S7 on the results of the conjunction analysis.

Supplementary Table S6. Resting state functional connectivity of left middle frontal gyrus (MFG): Interaction of SAD and ELA.

| **Anatomical region** | **Peak voxel MNI coordinates** | **clustersize** | **p (FDR)** |
| --- | --- | --- | --- |
| right inferior parietal but supramarginal and angular gyri, right angular gyrus, right supramarginal gyrus **(abbr. rIPL)** | 58, -50, 50 | 418 | p<0.001 |
| cerebellum | -38, -70, -30 | 364 | p<0.001 |
| right middle frontal gyrus, right precentral gyrus **(abbr. rMFG)** | 40, 16, 54 | 236 | p<0.001 |
| right lingual gyrus, left lingual gyrus, left calcarine fissure and surrounding cortex, left cuneus, right calcarine fissure and surrounding cortex, right superior occipital gyrus, right inferior occipital gyrus, right middle occipital gyrus, right middle temporal gyrus, right cuneus, left superior occipital gyrus, right fusiform gyrus, right inferior temporal gyrus | 06, -76, -04* | 5848 | p<0.001 |
| right postcentral gyrus, right precentral gyrus, left paracentral lobule, left postcentral gyrus, right paracentral lobule, left precentral gyrus, right supplementary motor area | 06, -26, 56* | 3282 | p<0.001 |
| left postcentral gyrus, left inferior parietal but supramarginal and angular gyri, left precentral gyrus, left rolandic operculum, left supramarginal gyrus | -42, -24, 36** | 604 | p<0.001 |

FDR= false discovery rate, voxel size 2x2x2mm^3^. *Cluster overlaps with connectivity pattern of both left calcarine fissure/ precuneus (CAL) and subgenual anterior cingulate cortex (sACC) clusters of MVPA. ** Cluster overlaps with connectivity pattern of CAL cluster from MVPA. Only abbreviations for the clusters that do not significantly overlap with the connectivity patterns of the other MVPA clusters are listed. The remaining abbreviations can be found in Table S7 on the results of the conjunction analysis.

Supplementary Table S7. Interaction of SAD and ELA: spatial convergence of the resting state connectivity patterns of MVPA clusters.

| **Conjunction** | **Anatomical region** | **Peak voxel MNI coordinates** | **Cluster size** | **p (FDR)** |
| --- | --- | --- | --- | --- |
| sACC and CAL | left superior temporal gyrus, left middle temporal gyrus **(abbr. lSTG/lMTG)** | -66, -22, 06 | 79 | p=0.036 |
|  | right rolandic operculum, right Heschl gyrus, right superior temporal gyrus, right Insula **(abbr. rROL)** | 36, -30, 18 | 136 | p=0.005 |
|  | left rolandic operculum, left Heschl gyrus, left Insula, left superior temporal gyrus, left supramarginal gyrus **(abbr. lROL)** | -30, -28, 14 | 133 | p=0.005 |
|  | left postcentral gyrus, left inferior parietal gyrus but supramarginal and angular gyri, left supramarginal gyrus **(abbr. lPoCG/lIPL2)** | -50, -16, 56 | 285 | p<0.001 |
| CAL and MFG | left postcentral gyrus, left inferior parietal gyrus but supramarginal and angular gyri, left supramarginal gyrus, left precentral gyrus **(abbr. lPoCG/lIPL1)** | -46, -26, 36 | 143 | p=0.003 |
|  | left postcentral gyrus, left precentral gyrus **(abbr. lPoCG/lPreCG)** | -58, -12, 36 | 141 | p=0.003 |
| sACC, CAL, MFG | right lingual gyrus, left calcarine fissure and surrounding cortex, left cuneus, left lingual gyrus, right calcarine fissure and surrounding cortex, left superior occipital gyrus, right fusiform gyrus **(abbr. rLING)*** | 12, -52, -02 | 2985 | p<0.001 |
|  | left paracentral lobule, left postcentral gyrus, left precentral gyrus, right paracentral lobule **(abbr. lPCL)** | -12, -22, 78 | 375 | p<0.001 |
|  | right inferior temporal gyrus, right middle occipital gyrus, right middle temporal gyrus, right inferior occipital gyrus **(abbr. rITG)*** | 48, -68, -08 | 256 | p<0.001 |
|  | right superior occipital gyrus, right cuneus, right middle occipital gyrus **(abbr. rSOG)*** | 24, -78, 28 | 232 | p<0.001 |
|  | right inferior occipital gyrus, right middle occipital gyrus, right lingual gyrus, right fusiform gyrus **(abbr. rIOG)*** | 34, -84, -14 | 100 | p=0.018 |
|  | right precentral gyrus, right postcentral gyrus **(abbr. rPreCG)** | 50, -14, 46 | 96 | p=0.018 |

CAL= left calcarine fissure/ precuneus, MFG= left middle frontal gyrus, sACC= subgenual anterior cingulate cortex, FDR= false discovery rate, voxel size 2x2x2mm^3^. Clusters marked with an asterisk emerged from the conjunction of identical clusters and were treated as single functional unit under the label “right occipital cluster”.

Supplementary Table S8. Post hoc comparison of functional connectivity between healthy controls and individuals with SAD under conditions of low and high ELA.

**low ELA**

| **Connection** | **r (Ce)** | **r (Se)** | **T(df)** | **p** |
| --- | --- | --- | --- | --- |
| CAL to left CAU 1 | 0.0962 | 0.1815 | T(77)=3.2 | p=0.002 |
| CAL to left PCL | -0.0275 | -0.1068 | T(77)=-2.6 | p=0.01 |
| CAL to left PoCG/ IPL 1 | -0.0424 | -0.1109 | T(77)=-2.8 | p=0.006 |
| CAL to left PoCG/ IPL 2 | -0.0530 | -0.1147 | T(77)=-2.1 | p=0.043 |
| CAL to left PoCG/ PreCG | -0.0315 | -0.0956 | T(77)=-1.9 | p=0.057 |
| CAL to left PreCG | -0.0683 | -0.1145 | T(77)=-2.1 | p=0.043 |
| CAL to left STG/ MTG | -0.0310 | -0.1149 | T(77)=-3.4 | **p<0.001*** |
| CAL to left ROL | -0.0360 | -0.1064 | T(77)=-2.8 | p=0.007 |
| CAL to right occipital cluster | -0.0015 | -0.1081 | T(77)=-2.7 | p=0.010 |
| CAL to right CAU 1 | 0.0684 | 0.1238 | T(77)=2.5 | p=0.016 |
| CAL to right PreCG | -0.0349 | -0.1114 | T(77)=-2.2 | p=0.032 |
| CAL to right ROL | -0.0351 | -0.1218 | T(77)=-3.2 | p=0.002 |
| MFG to Cerebellum | 0.0809 | -0.0060 | T(77)=-3.6 | **p<0.001*** |
| MFG to left PCL | -0.0378 | 0.0220 | T(77)=2.5 | p=0.015 |
| MFG to left PoCG/ IPL 1 | -0.0236 | 0.0509 | T(77)=3.6 | **p<0.001*** |
| MFG to left PoCG/ PreCG | -0.0096 | 0.0167 | T(77)=1.1 | p=0.267 |
| MFG to right occipital cluster | -0.0403 | 0.0077 | T(77)=2.2 | p=0.028 |
| MFG to right IPL | 0.1196 | -0.0074 | T(77)=-3.9 | **p<0.001*** |
| MFG to right MFG | 0.1782 | 0.0666 | T(77)=-3.4 | **p=0.001*** |
| MFG to right PreCG | -0.0210 | 0.0338 | T(77)=2.2 | p=0.032 |
| sACC to left CAU 2 | -0.0187 | 0.0461 | T(77)=3.6 | **p<0.001*** |
| sACC to left HIP | 0.1070 | 0.0774 | T(77)=-0.9 | p=0.376 |
| sACC to left ORB | 0.1498 | 0.0840 | T(77)=-1.9 | p=0.064 |
| sACC to left PCL | -0.0062 | -0.0416 | T(77)=-1.8 | p=0.082 |
| sACC to left PoCG/ IPL 2 | -0.0107 | -0.0492 | T(77)=-1.7 | p=0.101 |
| sACC to left SFG | 0.0439 | -0.0217 | T(77)=-2.4 | p=0.018 |
| sACC to left STG/ MTG | 0.0080 | -0.0108 | T(77)=-0.9 | p=0.385 |
| sACC to left STG/ MTG/ SMG | 0.0010 | -0.0642 | T(77)=-3.4 | **p=0.001*** |
| sACC to left THA | -0.0287 | 0.0192 | T(77)=3.6 | **p<0.001*** |
| sACC to left ROL | 0.0156 | -0.0174 | T(77)=-1.8 | p=0.083 |
| sACC to right occipital cluster | -0.0091 | -0.0615 | T(77)=-2.4 | p=0.017 |
| sACC to right CAU 2 | -0.0404 | 0.0321 | T(77)=4.9 | **p<0.001*** |
| sACC to right MTG | 0.0614 | -0.0072 | T(77)=-2.4 | p=0.021 |
| sACC to right PCG | -0.0040 | 0.0569 | T(77)=3.4 | **p=0.001*** |
| sACC to right PreCG | 0.0032 | -0.0626 | T(77)=-2.5 | p=0.015 |
| sACC to right ROL | 0.0171 | -0.0159 | T(77)=-1.5 | p=0.137 |

**high ELA**

| **Connection** | **r (CE)** | **r (SE)** | **T(df)** | **p** |
| --- | --- | --- | --- | --- |
| CAL to left CAU 1 | 0.2703 | 0.1147 | T(39)=-3.5 | **p=0.001*** |
| CAL to left PCL | -0.1919 | -0.0110 | T(39)=3.5 | **p=0.001*** |
| CAL to left PoCG/ IPL 1 | -0.1552 | -0.0087 | T(39)=4.1 | **p<0.001*** |
| CAL to left PoCG/ IPL 2 | -0.1836 | -0.0185 | T(39)=3.8 | **p<0.001*** |
| CAL to left PoCG/ PreCG | -0.1990 | -0.0363 | T(39)=3.7 | **p<0.001*** |
| CAL to left PreCG | -0.1698 | -0.0288 | T(39)=4.4 | **p<0.001*** |
| CAL to left STG/ MTG | -0.1376 | -0.0050 | T(39)=3.1 | p=0.004 |
| CAL to left ROL | -0.1687 | -0.0238 | T(39)=3.5 | **p=0.001*** |
| CAL to right occipital cluster | -0.2438 | -0.0169 | T(39)=5.1 | **p<0.001*** |
| CAL to right CAU 1 | 0.1494 | 0.0009 | T(39)=-4.3 | **p<0.001*** |
| CAL to right PreCG | -0.1935 | -0.0207 | T(39)=3.9 | **p<0.001*** |
| CAL to right ROL | -0.1622 | -0.0211 | T(39)=3.3 | p=0.002 |
| MFG to Cerebellum | 0.0055 | 0.1412 | T(39)=4.3 | **p<0.001*** |
| MFG to left PCL | 0.0566 | -0.0934 | T(39)=-5.2 | **p<0.001*** |
| MFG to left PoCG/ IPL 1 | 0.0503 | -0.0772 | T(39)=-4.9 | **p<0.001*** |
| MFG to left PoCG/ PreCG | 0.0759 | -0.0856 | T(39)=-4.8 | **p<0.001*** |
| MFG to right occipital cluster | 0.0412 | -0.1173 | T(39)=-5.3 | **p<0.001*** |
| MFG to right IPL | 0.0136 | 0.1694 | T(39)=3.2 | p=0.003 |
| MFG to right MFG | 0.0696 | 0.2428 | T(39)=3.3 | p=0.002 |
| MFG to right PreCG | 0.0621 | -0.0788 | T(39)=-3.9 | **p<0.001*** |
| sACC to left CAU 2 | 0.0766 | -0.0285 | T(39)=-3.5 | **p=0.001*** |
| sACC to left HIP | -0.0330 | 0.1757 | T(39)=5.4 | **p<0.001*** |
| sACC to left ORB | -0.0179 | 0.1609 | T(39)=4.1 | **p<0.001*** |
| sACC to left PCL | -0.1033 | 0.0588 | T(39)=5.1 | **p<0.001*** |
| sACC to left PoCG/ IPL 2 | -0.0918 | 0.0677 | T(39)=5.0 | **p<0.001*** |
| sACC to left SFG | -0.0745 | 0.0704 | T(39)=4.8 | **p<0.001*** |
| sACC to left STG/ MTG | -0.0795 | 0.0973 | T(39)=7.1 | **p<0.001*** |
| sACC to left STG/ MTG/ SMG | -0.0617 | 0.0518 | T(39)=5.1 | **p<0.001*** |
| sACC to left THA | 0.0491 | -0.0674 | T(39)=-4.8 | **p<0.001*** |
| sACC to left ROL | -0.0639 | 0.0850 | T(39)=6.0 | **p<0.001*** |
| sACC to right occipital cluster | -0.0934 | 0.0697 | T(39)=5.2 | **p<0.001*** |
| sACC to right CAU 2 | 0.0511 | -0.0278 | T(39)=-3.1 | p=0.004 |
| sACC to right MTG | -0.0472 | 0.1006 | T(39)=4.1 | **p<0.001*** |
| sACC to right PCG | 0.0611 | -0.0431 | T(39)=-4.3 | **p<0.001*** |
| sACC to right PreCG | -0.0722 | 0.0727 | T(39)=4.4 | **p<0.001*** |
| sACC to right ROL | -0.0647 | 0.0960 | T(39)=6.3 | **p<0.001*** |

T-tests of the Z-transformed bivariate correlation coefficients of the connections of the network. Ce: control participants with low ELA levels, CE: control participants with high ELA levels, Se: participants with SAD and low ELA levels, SE: participants with SAD and high ELA levels. For abbreviations of anatomical structures, see Fig. 3 and Tables S4-7. * after Bonferroni correction for multiple testing for 36 connections significant for p<0.05.

Supplementary Table S9. Post hoc comparison of functional connectivity - absolute functional connectivity for healthy controls and individuals with SAD under conditions of low and high ELA.

**Individuals without SAD**

| **Connection** | **Ce** | | | **CE** | | |
| --- | --- | --- | --- | --- | --- | --- |
|  | **r (Ce)** | **T(df)** | **p** | **r(CE)** | **T(df)** | **p** |
| CAL to left CAU 1 | 0.0962 | T(48)=5.8 | **p<0.001*** | 0.2703 | T(21)=8.4 | **p<0.001*** |
| CAL to left PCL | -0.0275 | T(48)=-1.4 | p=0.162 | -0.1919 | T(21)=-4.7 | **p<0.001*** |
| CAL to left PoCG/ IPL 1 | -0.0424 | T(48)=-2.8 | p=0.008 | -0.1552 | T(21)=-5.9 | **p<0.001*** |
| CAL to left PoCG/ IPL 2 | -0.0530 | T(48)=-2.7 | p=0.009 | -0.1836 | T(21)=-5.9 | **p<0.001*** |
| CAL to left PoCG/ PreCG | -0.0315 | T(48)=-1.5 | p=0.152 | -0.1990 | T(21)=-6.7 | **p<0.001*** |
| CAL to left PreCG | -0.0683 | T(48)=-4.8 | **p<0.001*** | -0.1698 | T(21)=-7.1 | **p<0.001*** |
| CAL to left STG/ MTG | -0.0310 | T(48)=-2.0 | p=0.054 | -0.1376 | T(21)=-4.6 | **p<0.001*** |
| CAL to left ROL | -0.0360 | T(48)=-2.2 | p=0.029 | -0.1687 | T(21)=-5.9 | **p<0.001*** |
| CAL to right occipital cluster | -0.0015 | T(48)=0.0 | p=0.964 | -0.2438 | T(21)=-7.7 | **p<0.001*** |
| CAL to right CAU 1 | 0.0684 | T(48)=4.8 | **p<0.001*** | 0.1494 | T(21)=6.5 | **p<0.001*** |
| CAL to right PreCG | -0.0349 | T(48)=-1.5 | p=0.147 | -0.1935 | T(21)=-6.1 | **p<0.001*** |
| CAL to right ROL | -0.0351 | T(48)=-2.1 | p=0.039 | -0.1622 | T(21)=-7.0 | **p<0.001*** |
| MFG to Cerebellum | 0.0809 | T(48)=5.5 | **p<0.001*** | 0.0055 | T(21)=0.3 | p=0.740 |
| MFG to left PCL | -0.0378 | T(48)=-2.5 | p=0.016 | 0.0566 | T(21)=3.1 | p=0.006 |
| MFG to left PoCG/ IPL 1 | -0.0236 | T(48)=-1.7 | p=0.094 | 0.0503 | T(21)=3.0 | p=0.008 |
| MFG to left PoCG/ PreCG | -0.0096 | T(48)=-0.6 | p=0.544 | 0.0759 | T(21)=3.5 | p=0.002 |
| MFG to right occipital cluster | -0.0403 | T(48)=-2.9 | p=0.005 | 0.0412 | T(21)=2.1 | p=0.051 |
| MFG to right IPL | 0.1196 | T(48)=5.8 | **p<0.001*** | 0.0136 | T(21)=0.4 | p=0.659 |
| MFG to right MFG | 0.1782 | T(48)=8.6 | **p<0.001*** | 0.0696 | T(21)=1.9 | p=0.066 |
| MFG to right PreCG | -0.0210 | T(48)=-1.3 | p=0.199 | 0.0621 | T(21)=2.6 | p=0.018 |
| sACC to left CAU 2 | -0.0187 | T(48)=-1.9 | p=0.061 | 0.0766 | T(21)=3.8 | **p=0.001*** |
| sACC to left HIP | 0.1070 | T(48)=4.8 | **p<0.001*** | -0.0330 | T(21)=-1.2 | p=0.249 |
| sACC to left ORB | 0.1498 | T(48)=6.6 | **p<0.001*** | -0.0179 | T(21)=-0.6 | p=0.524 |
| sACC to left PCL | -0.0062 | T(48)=-0.5 | p=0.635 | -0.1033 | T(21)=-4.6 | **p<0.001*** |
| sACC to left PoCG/ IPL 2 | -0.0107 | T(48)=-0.7 | p=0.481 | -0.0918 | T(21)=-3.9 | **p<0.001*** |
| sACC to left SFG | 0.0439 | T(48)=2.8 | p=0.007 | -0.0745 | T(21)=-3.0 | p=0.006 |
| sACC to left STG/ MTG | 0.0080 | T(48)=0.6 | p=0.557 | -0.0795 | T(21)=-4.7 | **p<0.001*** |
| sACC to left STG/ MTG/ SMG | 0.0010 | T(48)=0.1 | p=0.936 | -0.0617 | T(21)=-4.5 | **p<0.001*** |
| sACC to left THA | -0.0287 | T(48)=-4.9 | **p<0.001*** | 0.0491 | T(21)=3.2 | p=0.004 |
| sACC to left ROL | 0.0156 | T(48)=1.4 | p=0.163 | -0.0639 | T(21)=-4.9 | **p<0.001*** |
| sACC to right occipital cluster | -0.0091 | T(48)=-0.7 | p=0.493 | -0.0934 | T(21)=-4.7 | **p<0.001*** |
| sACC to right CAU 2 | -0.0404 | T(48)=-5.4 | **p<0.001*** | 0.0511 | T(21)=3.6 | p=0.002 |
| sACC to right MTG | 0.0614 | T(48)=3.4 | p=0.001 | -0.0472 | T(21)=-1.8 | p=0.094 |
| sACC to right PCG | -0.0040 | T(48)=-0.4 | p=0.697 | 0.0611 | T(21)=3.8 | **p=0.001*** |
| sACC to right PreCG | 0.0032 | T(48)=0.2 | p=0.853 | -0.0722 | T(21)=-3.3 | p=0.004 |
| sACC to right ROL | 0.0171 | T(48)=1.4 | p=0.160 | -0.0647 | T(21)=-4.5 | **p<0.001*** |

**Individuals with SAD**

| **Connection** | **Se** | | | **SE** | | |
| --- | --- | --- | --- | --- | --- | --- |
|  | **r(Se)** | **T(df)** | **p** | **r(SE)** | **T(df)** | **p** |
| CAL to left CAU 1 | 0.1815 | T(29)=8.8 | **p<0.001*** | 0.1147 | T(18)=3.8 | p=0.001 |
| CAL to left PCL | -0.1068 | T(29)=-4.9 | **p<0.001*** | -0.0110 | T(18)=-0.4 | p=0.709 |
| CAL to left PoCG/ IPL 1 | -0.1109 | T(29)=-5.9 | **p<0.001*** | -0.0087 | T(18)=-0.4 | p=0.717 |
| CAL to left PoCG/ IPL 2 | -0.1147 | T(29)=-5.4 | **p<0.001*** | -0.0185 | T(18)=-0.6 | p=0.547 |
| CAL to left PoCG/ PreCG | -0.0956 | T(29)=-4.1 | **p<0.001*** | -0.0363 | T(18)=-1.1 | p=0.288 |
| CAL to left PreCG | -0.1145 | T(29)=-6.8 | **p<0.001*** | -0.0288 | T(18)=-1.4 | p=0.167 |
| CAL to left STG/ MTG | -0.1149 | T(29)=-6.5 | **p<0.001*** | -0.0050 | T(18)=-0.2 | p=0.871 |
| CAL to left ROL | -0.1064 | T(29)=-5.5 | **p<0.001*** | -0.0238 | T(18)=-0.8 | p=0.422 |
| CAL to right occipital cluster | -0.1081 | T(29)=-4.8 | **p<0.001*** | -0.0169 | T(18)=-0.5 | p=0.593 |
| CAL to right CAU 1 | 0.1238 | T(29)=7.3 | **p<0.001*** | 0.0009 | T(18)=0.0 | p=0.974 |
| CAL to right PreCG | -0.1114 | T(29)=-4.9 | **p<0.001*** | -0.0207 | T(18)=-0.7 | p=0.516 |
| CAL to right ROL | -0.1218 | T(29)=-5.4 | **p<0.001*** | -0.0211 | T(18)=-0.6 | p=0.573 |
| MFG to Cerebellum | -0.0060 | T(29)=-0.3 | p=0.760 | 0.1412 | T(18)=5.0 | **p<0.001*** |
| MFG to left PCL | 0.0220 | T(29)=1.2 | p=0.234 | -0.0934 | T(18)=-4.1 | **p<0.001*** |
| MFG to left PoCG/ IPL 1 | 0.0509 | T(29)=3.6 | **p=0.001*** | -0.0772 | T(18)=-3.9 | **p<0.001*** |
| MFG to left PoCG/ PreCG | 0.0167 | T(29)=1.1 | p=0.294 | -0.0856 | T(18)=-3.2 | p=0.005 |
| MFG to right occipital cluster | 0.0077 | T(29)=0.5 | p=0.628 | -0.1173 | T(18)=-5.3 | **p<0.001*** |
| MFG to right IPL | -0.0074 | T(29)=-0.3 | p=0.773 | 0.1694 | T(18)=4.3 | **p<0.001*** |
| MFG to right MFG | 0.0666 | T(29)=2.7 | p=0.011 | 0.2428 | T(18)=6.4 | **p<0.001*** |
| MFG to right PreCG | 0.0338 | T(29)=1.9 | p=0.072 | -0.0788 | T(18)=-3.0 | p=0.008 |
| sACC to left CAU 2 | 0.0461 | T(29)=2.8 | p=0.008 | -0.0285 | T(18)=-1.3 | p=0.216 |
| sACC to left HIP | 0.0774 | T(29)=3.5 | p=0.002 | 0.1757 | T(18)=6.7 | **p<0.001*** |
| sACC to left ORB | 0.0840 | T(29)=3.4 | p=0.002 | 0.1609 | T(18)=4.8 | **p<0.001*** |
| sACC to left PCL | -0.0416 | T(29)=-2.9 | p=0.007 | 0.0588 | T(18)=2.6 | p=0.017 |
| sACC to left PoCG/ IPL 2 | -0.0492 | T(29)=-3.0 | p=0.005 | 0.0677 | T(18)=3.3 | p=0.004 |
| sACC to left SFG | -0.0217 | T(29)=-0.9 | p=0.359 | 0.0704 | T(18)=4.0 | **p<0.001*** |
| sACC to left STG/ MTG | -0.0108 | T(29)=-0.7 | p=0.514 | 0.0973 | T(18)=5.3 | **p<0.001*** |
| sACC to left STG/ MTG/ SMG | -0.0642 | T(29)=-5.0 | **p<0.001*** | 0.0518 | T(18)=2.8 | p=0.011 |
| sACC to left THA | 0.0192 | T(29)=1.6 | p=0.122 | -0.0674 | T(18)=-3.5 | p=0.003 |
| sACC to left ROL | -0.0174 | T(29)=-1.1 | p=0.283 | 0.0850 | T(18)=4.1 | **p<0.001*** |
| sACC to right occipital cluster | -0.0615 | T(29)=-3.6 | **p<0.001*** | 0.0697 | T(18)=2.8 | p=0.013 |
| sACC to right CAU 2 | 0.0321 | T(29)=2.3 | p=0.032 | -0.0278 | T(18)=-1.3 | p=0.225 |
| sACC to right MTG | -0.0072 | T(29)=-0.3 | p=0.749 | 0.1006 | T(18)=4.5 | **p<0.001*** |
| sACC to right PCG | 0.0569 | T(29)=3.6 | **p=0.001*** | -0.0431 | T(18)=-2.4 | p=0.028 |
| sACC to right PreCG | -0.0626 | T(29)=-3.3 | p=0.002 | 0.0727 | T(18)=2.9 | p=0.009 |
| sACC to right ROL | -0.0159 | T(29)=-0.8 | p=0.436 | 0.0960 | T(18)=4.4 | **p<0.001*** |

T-tests of the Z-transformed bivariate correlation coefficients of the connections of the network. Ce: control participants with low ELA levels, CE: control participants with high ELA levels, Se: participants with SAD and low ELA levels, SE: participants with SAD and high ELA levels. For abbreviations of anatomical structures, see Fig. 3 and Tables S4-7. *after Bonferroni correction for multiple testing for 36 connections significant for p<0.05.

Supplementary Table S10. Association of functional connectivity with severity of ELA for healthy controls and individuals with SAD.

|  |  | CAL to left CAU 1 | CAL to left PCL | CAL to left PoCG/ IPL 1 | CAL to left PoCG/ IPL 2 | CAL to left PoCG/ PreCG | CAL to left PreCG | CAL to left STG/ MTG | CAL to left ROL | CAL to right occipital cluster | CAL to right CAU 1 | CAL to right PreCG | CAL to right ROL | MFG to Cerebellum | MFG to left PCL |
| --- | --- | --- | --- | --- | --- | --- | --- | --- | --- | --- | --- | --- | --- | --- | --- |
| **CTQ subscales** | |  |  |  |  |  |  |  |  |  |  |  |  |  |  |
| **emotional abuse** | **r (no SAD)** | 0.4798 | -0.3857 | -0.3582 | -0.3778 | -0.3436 | -0.2956 | -0.3614 | -0.3752 | -0.3487 | 0.2907 | -0.3656 | -0.4073 | -0.2602 | 0.2779 |
|  | **p (no SAD)** | p<0.001 | p<0.001 | p=0.002 | p=0.001 | p=0.003 | p=0.012 | p=0.002 | p=0.001 | p=0.003 | p=0.014 | p=0.002 | p<0.001 | p=0.028 | p=0.019 |
|  | **r (SAD)** | -0.4220 | 0.3718 | 0.4591 | 0.4257 | 0.3166 | 0.3767 | 0.5564 | 0.4271 | 0.4355 | -0.5111 | 0.4701 | 0.4415 | 0.4641 | -0.3284 |
|  | **p (SAD)** | p=0.003 | p=0.01 | p<0.001 | p=0.002 | p=0.027 | p=0.008 | p<0.001 | p=0.002 | p=0.002 | p<0.001 | p<0.001 | p=0.001 | p<0.001 | p=0.021 |
|  | **z** | 5.0960 | -4.1761 | -4.5624 | -4.4636 | -3.5936 | -3.6714 | -5.2701 | -4.4567 | -4.3510 | 4.5234 | -4.6805 | -4.7482 | -4.0274 | 3.2814 |
|  | **p** | **p<0.001*** | **p<0.001*** | **p<0.001*** | **p<0.001*** | **p<0.001*** | **p<0.001*** | **p<0.001*** | **p<0.001*** | **p<0.001*** | **p<0.001*** | **p<0.001*** | **p<0.001*** | **p<0.001*** | p=0.001 |
| **physical abuse** | **r (no SAD)** | 0.0343 | 0.0095 | 0.0384 | 0.0452 | 0.0377 | 0.0259 | 0.0557 | 0.0024 | -0.0233 | 0.1486 | 0.0303 | -0.0026 | -0.0821 | 0.0414 |
|  | **p (no SAD)** | p=0.776 | p=0.937 | p=0.751 | p=0.708 | p=0.755 | p=0.830 | p=0.645 | p=0.984 | p=0.847 | p=0.216 | p=0.802 | p=0.983 | p=0.496 | p=0.732 |
|  | **r (SAD)** | -0.4158 | 0.3818 | 0.4667 | 0.4164 | 0.3708 | 0.2836 | 0.5903 | 0.4383 | 0.4456 | -0.3488 | 0.4546 | 0.4440 | 0.2955 | -0.2167 |
|  | **p (SAD)** | p=0.003 | p=0.007 | p<0.001 | p=0.003 | p=0.009 | p=0.048 | p<0.001 | p=0.002 | p=0.001 | p=0.014 | p=0.001 | p=0.001 | p=0.039 | p=0.135 |
|  | **z** | 2.4982 | -2.0569 | -2.4485 | -2.0853 | -1.8419 | -1.3917 | -3.2601 | -2.4500 | -2.6322 | 2.6913 | -2.4105 | -2.5133 | -2.0265 | 1.3704 |
|  | **p** | p=0.012 | p=0.040 | p=0.014 | p=0.037 | p=0.065 | p=0.164 | p=0.001 | p=0.014 | p=0.008 | p=0.007 | p=0.015 | p=0.012 | p=0.043 | p=0.171 |
| **sexual abuse** | **r (no SAD)** | 0.1904 | -0.0270 | -0.1335 | -0.0855 | -0.1438 | -0.1103 | -0.2702 | -0.1309 | -0.0943 | 0.1361 | -0.0850 | -0.1539 | -0.0832 | 0.0278 |
|  | **p (no SAD)** | p=0.112 | p=0.823 | p=0.267 | p=0.478 | p=0.232 | p=0.360 | p=0.023 | p=0.277 | p=0.434 | p=0.258 | p=0.480 | p=0.200 | p=0.491 | p=0.818 |
|  | **r (SAD)** | -0.1936 | 0.2999 | 0.3004 | 0.2188 | 0.3845 | 0.3357 | 0.4847 | 0.3560 | 0.3879 | -0.1503 | 0.3177 | 0.3954 | 0.2969 | -0.1037 |
|  | **p (SAD)** | p=0.183 | p=0.036 | p=0.036 | p=0.131 | p=0.006 | p=0.018 | p<0.001 | p=0.012 | p=0.006 | p=0.303 | p=0.026 | p=0.005 | p=0.038 | p=0.478 |
|  | **z** | 2.0367 | -1.7622 | -2.3271 | -1.6139 | -2.8817 | -2.4095 | -4.2230 | -2.6398 | -2.6396 | 1.5107 | -2.1701 | -3.0031 | -2.0403 | 0.6908 |
|  | **p** | p=0.042 | p=0.078 | p=0.020 | p=0.107 | p=0.004 | p=0.016 | **p<0.001*** | p=0.008 | p=0.008 | p=0.131 | p=0.030 | p=0.003 | p=0.041 | p=0.490 |
| **emotional neglect** | **r (no SAD)** | 0.4305 | -0.4079 | -0.3712 | -0.3581 | -0.3439 | -0.3508 | -0.3331 | -0.4296 | -0.4043 | 0.2491 | -0.3586 | -0.4096 | -0.3341 | 0.2985 |
|  | **p (no SAD)** | p<0.001 | p<0.001 | p=0.001 | p=0.002 | p=0.003 | p=0.003 | p=0.005 | p<0.001 | p<0.001 | p=0.036 | p=0.002 | p<0.001 | p=0.004 | p=0.011 |
|  | **r (SAD)** | -0.2760 | 0.4904 | 0.4985 | 0.5222 | 0.3186 | 0.4543 | 0.5205 | 0.4888 | 0.4307 | -0.5771 | 0.5149 | 0.4549 | 0.3633 | -0.3326 |
|  | **p (SAD)** | p=0.055 | p<0.001 | p<0.001 | p<0.001 | p=0.026 | p=0.001 | p<0.001 | p<0.001 | p=0.002 | p<0.001 | p<0.001 | p=0.001 | p=0.010 | p=0.020 |
|  | **z** | 3.8965 | -5.0793 | -4.9088 | -4.9976 | -3.6070 | -4.4863 | -4.8366 | -5.2062 | -4.6595 | 4.7801 | -4.9483 | -4.8505 | -3.8140 | 3.4238 |
|  | **p** | **p<0.001*** | **p<0.001*** | **p<0.001*** | **p<0.001*** | **p<0.001*** | **p<0.001*** | **p<0.001*** | **p<0.001*** | **p<0.001*** | **p<0.001*** | **p<0.001*** | **p<0.001*** | **p<0.001*** | p<0.001 |
| **physical neglect** | **r (no SAD)** | 0.3969 | -0.3534 | -0.3183 | -0.2981 | -0.3042 | -0.3685 | -0.2227 | -0.4126 | -0.3289 | 0.2033 | -0.2993 | -0.3680 | -0.3768 | 0.4334 |
|  | **p (no SAD)** | p<0.001 | p=0.003 | p=0.008 | p=0.012 | p=0.010 | p=0.002 | p=0.062 | p<0.001 | p=0.005 | p=0.089 | p=0.011 | p=0.002 | p=0.001 | p<0.001 |
|  | **r (SAD)** | -0.2730 | 0.4746 | 0.5109 | 0.4796 | 0.3596 | 0.3717 | 0.5073 | 0.4810 | 0.4110 | -0.4369 | 0.4855 | 0.4565 | 0.3003 | -0.3092 |
|  | **p (SAD)** | p=0.058 | p<0.001 | p<0.001 | p<0.001 | p=0.011 | p=0.009 | p<0.001 | p<0.001 | p=0.003 | p=0.002 | p<0.001 | p<0.001 | p=0.036 | p=0.031 |
|  | **z** | 3.6671 | -4.6374 | -4.6814 | -4.3472 | -3.6173 | -4.0705 | -4.1150 | -5.0445 | -4.0774 | 3.5335 | -4.3943 | -4.6043 | -3.6991 | 4.1054 |
|  | **p** | **p<0.001*** | **p<0.001*** | **p<0.001*** | **p<0.001*** | **p<0.001*** | **p<0.001*** | **p<0.001*** | **p<0.001*** | **p<0.001*** | **p<0.001*** | **p<0.001*** | **p<0.001*** | **p<0.001*** | **p<0.001*** |
| **total CTQ score** | **r (no SAD)** | 0.4947 | -0.4158 | -0.3889 | -0.3792 | -0.3688 | -0.3669 | -0.3632 | -0.4482 | -0.4060 | 0.3028 | -0.3769 | -0.4436 | -0.3578 | 0.3503 |
|  | **p (no SAD)** | p<0.001 | p<0.001 | p<0.001 | p=0.001 | p=0.002 | p=0.002 | p=0.002 | p<0.001 | p<0.001 | p=0.010 | p=0.001 | p<0.001 | p=0.002 | p=0.003 |
|  | **r (SAD)** | -0.3743 | 0.4982 | 0.5441 | 0.5104 | 0.4182 | 0.4611 | 0.6410 | 0.5358 | 0.5125 | -0.5183 | 0.5507 | 0.5331 | 0.4299 | -0.3251 |
|  | **p (SAD)** | p=0.008 | p<0.001 | p<0.001 | p<0.001 | p=0.003 | p<0.001 | p<0.001 | p<0.001 | p<0.001 | p<0.001 | p<0.001 | p<0.001 | p=0.002 | p=0.023 |
|  | **z** | 4.9013 | -5.1833 | -5.3454 | -5.0412 | -4.3610 | -4.6282 | -5.9738 | -5.6609 | -5.2221 | 4.6442 | -5.3211 | -5.6110 | -4.3694 | 3.6831 |
|  | **p** | **p<0.001*** | **p<0.001*** | **p<0.001*** | **p<0.001*** | **p<0.001*** | **p<0.001*** | **p<0.001*** | **p<0.001*** | **p<0.001*** | **p<0.001*** | **p<0.001*** | **p<0.001*** | **p<0.001*** | **p<0.001*** |

|  |  | MFG to left PoCG/ IPL 1 | MFG to left PoCG/ PreCG | MFG to right occipital cluster | MFG to right IPL | MFG to right MFG | MFG to right PreCG | sACC to left CAU 2 | sACC to left HIP | sACC to left ORB | sACC to left PCL | sACC to left PoCG/ IPL 2 | sACC to left SFG | sACC to left STG/ MTG | sACC to left STG/ MTG/ SMG |
| --- | --- | --- | --- | --- | --- | --- | --- | --- | --- | --- | --- | --- | --- | --- | --- |
| **CTQ subscales** | |  |  |  |  |  |  |  |  |  |  |  |  |  |  |
| **emotional abuse** | **r (no SAD)** | 0.2590 | 0.3306 | 0.3367 | -0.2463 | -0.2481 | 0.3193 | 0.3874 | -0.3190 | -0.3741 | -0.4361 | -0.3193 | -0.3170 | -0.2807 | -0.2038 |
|  | **p (no SAD)** | p=0.029 | p=0.005 | p=0.004 | p=0.038 | p=0.037 | p=0.007 | p<0.001 | p=0.007 | p=0.001 | p<0.001 | p=0.007 | p=0.007 | p=0.018 | p=0.088 |
|  | **r (SAD)** | -0.3991 | -0.4482 | -0.3068 | 0.4429 | 0.3934 | -0.3741 | -0.2070 | 0.2322 | 0.1288 | 0.4067 | 0.4148 | 0.1551 | 0.4114 | 0.4038 |
|  | **p (SAD)** | p=0.005 | p=0.001 | p=0.032 | p=0.001 | p=0.005 | p=0.008 | p=0.154 | p=0.108 | p=0.378 | p=0.004 | p=0.003 | p=0.287 | p=0.003 | p=0.004 |
|  | **z** | 3.6019 | 4.3265 | 3.4958 | -3.8097 | -3.5054 | 3.7927 | 3.2412 | -2.9703 | -2.7380 | -4.7094 | -4.0453 | -2.5388 | -3.8015 | -3.3256 |
|  | **p** | **p<0.001*** | **p<0.001*** | **p<0.001*** | **p<0.001*** | **p<0.001*** | **p<0.001*** | p=0.001 | p=0.003 | p=0.006 | **p<0.001*** | **p<0.001*** | p=0.011 | **p<0.001*** | p<0.001 |
| **physical abuse** | **r (no SAD)** | 0.0202 | 0.1245 | 0.0590 | -0.1470 | -0.1902 | 0.0809 | 0.0712 | -0.2347 | -0.1915 | -0.0869 | 0.1022 | -0.1267 | -0.0900 | -0.0707 |
|  | **p (no SAD)** | p=0.867 | p=0.301 | p=0.625 | p=0.221 | p=0.112 | p=0.502 | p=0.555 | p=0.049 | p=0.110 | p=0.470 | p=0.396 | p=0.292 | p=0.455 | p=0.558 |
|  | **r (SAD)** | -0.2426 | -0.3074 | -0.2323 | 0.2291 | 0.2166 | -0.2077 | -0.0449 | 0.0915 | 0.0195 | 0.1075 | 0.0585 | 0.1986 | 0.1232 | 0.1040 |
|  | **p (SAD)** | p=0.093 | p=0.032 | p=0.108 | p=0.113 | p=0.135 | p=0.152 | p=0.759 | p=0.532 | p=0.894 | p=0.462 | p=0.690 | p=0.171 | p=0.399 | p=0.477 |
|  | **z** | 1.4025 | 2.3196 | 1.5489 | -1.9974 | -2.1614 | 1.5287 | 0.6089 | -1.7334 | -1.1178 | -1.0216 | 0.2304 | -1.7216 | -1.1214 | -0.9177 |
|  | **p** | p=0.161 | p=0.020 | p=0.121 | p=0.046 | p=0.031 | p=0.126 | p=0.543 | p=0.083 | p=0.264 | p=0.307 | p=0.818 | p=0.085 | p=0.262 | p=0.359 |
| **sexual abuse** | **r (no SAD)** | 0.0492 | 0.1360 | 0.2614 | -0.0431 | -0.0646 | 0.0901 | -0.0684 | -0.1816 | -0.0962 | 0.0989 | 0.0762 | 0.0282 | -0.0215 | 0.0015 |
|  | **p (no SAD)** | p=0.684 | p=0.258 | p=0.028 | p=0.721 | p=0.592 | p=0.455 | p=0.571 | p=0.130 | p=0.425 | p=0.412 | p=0.527 | p=0.816 | p=0.859 | p=0.990 |
|  | **r (SAD)** | -0.1619 | -0.2339 | -0.3492 | 0.2232 | 0.1746 | -0.0379 | -0.0131 | 0.0874 | -0.1069 | 0.2557 | 0.1897 | 0.0949 | 0.1361 | 0.0441 |
|  | **p (SAD)** | p=0.266 | p=0.106 | p=0.014 | p=0.123 | p=0.230 | p=0.796 | p=0.929 | p=0.550 | p=0.465 | p=0.076 | p=0.192 | p=0.517 | p=0.351 | p=0.763 |
|  | **z** | 1.1135 | 1.9652 | 3.3113 | -1.4151 | -1.2629 | 0.6719 | -0.2902 | -1.4209 | 0.0566 | -0.8500 | -0.6059 | -0.3508 | -0.8300 | -0.2233 |
|  | **p** | p=0.265 | p=0.049 | p<0.001 | p=0.157 | p=0.207 | p=0.502 | p=0.772 | p=0.155 | p=0.955 | p=0.395 | p=0.545 | p=0.726 | p=0.407 | p=0.823 |
| **emotional neglect** | **r (no SAD)** | 0.2867 | 0.2447 | 0.3121 | -0.3320 | -0.3062 | 0.3093 | 0.4526 | -0.3695 | -0.4527 | -0.4425 | -0.2552 | -0.5031 | -0.3622 | -0.2770 |
|  | **p (no SAD)** | p=0.015 | p=0.040 | p=0.008 | p=0.005 | p=0.009 | p=0.009 | p<0.001 | p=0.002 | p<0.001 | p<0.001 | p=0.032 | p<0.001 | p=0.002 | p=0.019 |
|  | **r (SAD)** | -0.4406 | -0.3860 | -0.3320 | 0.5417 | 0.3763 | -0.4055 | -0.2196 | 0.2206 | 0.2341 | 0.3544 | 0.3750 | 0.2852 | 0.3490 | 0.3897 |
|  | **p (SAD)** | p=0.002 | p=0.006 | p=0.020 | p<0.001 | p=0.008 | p=0.004 | p=0.123 | p=0.128 | p=0.106 | p=0.012 | p=0.008 | p=0.047 | p=0.014 | p=0.006 |
|  | **z** | 4.0226 | 3.4407 | 3.4988 | -4.9848 | -3.7301 | 3.9286 | 3.7254 | -3.2065 | -3.8061 | -4.4305 | -3.4320 | -4.4356 | -3.8957 | -3.6451 |
|  | **p** | **p<0.001*** | p<0.001 | **p<0.001*** | **p<0.001*** | **p<0.001*** | **p<0.001*** | **p<0.001*** | p=0.001 | **p<0.001*** | **p<0.001*** | p<0.001 | **p<0.001*** | **p<0.001*** | **p<0.001*** |
| **physical neglect** | **r (no SAD)** | 0.3246 | 0.2942 | 0.4195 | -0.3894 | -0.2617 | 0.3610 | 0.3516 | -0.4160 | -0.3661 | -0.3604 | -0.1688 | -0.4065 | -0.3588 | -0.3247 |
|  | **p (no SAD)** | p=0.006 | p=0.013 | p<0.001 | p<0.001 | p=0.027 | p=0.002 | p=0.003 | p<0.001 | p=0.002 | p=0.002 | p=0.159 | p<0.001 | p=0.002 | p=0.006 |
|  | **r (SAD)** | -0.4149 | -0.4927 | -0.2767 | 0.5062 | 0.3202 | -0.3625 | -0.0146 | 0.2252 | 0.1808 | 0.3114 | 0.2930 | 0.2041 | 0.2378 | 0.2275 |
|  | **p (SAD)** | p=0.003 | p<0.001 | p=0.054 | p<0.001 | p=0.025 | p=0.010 | p=0.920 | p=0.120 | p=0.214 | p=0.029 | p=0.041 | p=0.160 | p=0.100 | p=0.116 |
|  | **z** | 4.0769 | 4.4146 | 3.8301 | -5.0742 | -3.1419 | 3.9695 | 2.0003 | -3.5199 | -2.9686 | -3.6638 | -2.4739 | -3.3442 | -3.2369 | -2.9776 |
|  | **p** | **p<0.001*** | **p<0.001*** | **p<0.001*** | **p<0.001*** | p=0.002 | **p<0.001*** | p=0.045 | **p<0.001*** | p=0.003 | **p<0.001*** | p=0.013 | p<0.001 | p=0.001 | p=0.003 |
| **total CTQ score** | **r (no SAD)** | 0.3122 | 0.3342 | 0.4032 | -0.3578 | -0.3241 | 0.3652 | 0.4355 | -0.4339 | -0.4664 | -0.4494 | -0.2552 | -0.4565 | -0.3681 | -0.2876 |
|  | **p (no SAD)** | p=0.008 | p=0.004 | p<0.001 | p=0.002 | p=0.006 | p=0.002 | p<0.001 | p<0.001 | p<0.001 | p<0.001 | p=0.032 | p<0.001 | p=0.002 | p=0.015 |
|  | **r (SAD)** | -0.4215 | -0.4566 | -0.3755 | 0.5003 | 0.3778 | -0.3565 | -0.1481 | 0.2200 | 0.1306 | 0.3753 | 0.3580 | 0.2369 | 0.3337 | 0.3192 |
|  | **p (SAD)** | p=0.002 | p<0.001 | p=0.008 | p<0.001 | p=0.007 | p=0.012 | p=0.310 | p=0.129 | p=0.371 | p=0.008 | p=0.012 | p=0.101 | p=0.019 | p=0.025 |
|  | **z** | 4.0465 | 4.4030 | 4.3072 | -4.8404 | -3.8433 | 3.9587 | 3.2260 | -3.6057 | -3.3357 | -4.6019 | -3.3292 | -3.8468 | -3.8407 | -3.2828 |
|  | **p** | **p<0.001*** | **p<0.001*** | **p<0.001*** | **p<0.001*** | **p<0.001*** | **p<0.001*** | **p<0.001*** | **p<0.001*** | **p<0.001*** | **p<0.001*** | **p<0.001*** | **p<0.001*** | **p<0.001*** | **p<0.001*** |
|  |  |  |  |  |  |  |  |  |  |  |  |  |  |  |  |

|  |  | sACC to left THA | sACC to left ROL | sACC to right occipital cluster | sACC to right CAU 2 | sACC to right MTG | sACC to right PCG | sACC to right PreCG | sACC to right ROL |  |  |  |  |  |  |
| --- | --- | --- | --- | --- | --- | --- | --- | --- | --- | --- | --- | --- | --- | --- | --- |
| **CTQ subscales** | |  |  |  |  |  |  |  |  |  |  |  | |  |  |
| **emotional abuse** | **r (no SAD)** | 0.4998 | -0.3530 | -0.4213 | 0.4966 | -0.2635 | 0.3738 | -0.2755 | -0.3184 |  |  |  |  |  |  |
|  | **p (no SAD)** | p<0.001 | p=0.003 | p<0.001 | p<0.001 | p=0.026 | p=0.001 | p=0.020 | p=0.007 |  |  |  |  |  |  |
|  | **r (SAD)** | -0.4628 | 0.3653 | 0.4226 | -0.2557 | 0.1933 | -0.4685 | 0.3967 | 0.3377 |  |  |  |  |  |  |
|  | **p (SAD)** | p<0.001 | p=0.010 | p=0.002 | p=0.076 | p=0.183 | p<0.001 | p=0.005 | p=0.018 |  |  |  |  |  |  |
|  | **z** | 5.4996 | -3.9384 | -4.7150 | 4.2235 | -2.4391 | 4.7195 | -3.6800 | -3.5691 |  |  |  |  |  |  |
|  | **p** | **p<0.001*** | **p<0.001*** | **p<0.001*** | **p<0.001*** | p=0.015 | **p<0.001*** | **p<0.001*** | **p<0.001*** |  |  |  |  |  |  |
| **physical abuse** | **r (no SAD)** | 0.2499 | -0.2073 | -0.0689 | 0.1692 | 0.0341 | 0.1222 | 0.0027 | -0.2027 |  |  |  |  |  |  |
|  | **p (no SAD)** | p=0.036 | p=0.083 | p=0.568 | p=0.158 | p=0.778 | p=0.310 | p=0.982 | p=0.090 |  |  |  |  |  |  |
|  | **r (SAD)** | -0.2211 | 0.0468 | 0.0929 | -0.0567 | 0.1517 | -0.1915 | 0.1302 | 0.0837 |  |  |  |  |  |  |
|  | **p (SAD)** | p=0.127 | p=0.750 | p=0.526 | p=0.699 | p=0.298 | p=0.188 | p=0.373 | p=0.567 |  |  |  |  |  |  |
|  | **z** | 2.5150 | -1.3472 | -0.8495 | 1.1922 | -0.6221 | 1.6590 | -0.6718 | -1.5162 |  |  |  |  |  |  |
|  | **p** | p=0.012 | p=0.178 | p=0.396 | p=0.233 | p=0.534 | p=0.097 | p=0.502 | p=0.129 |  |  |  |  |  |  |
| **sexual abuse** | **r (no SAD)** | -0.0410 | -0.0838 | 0.0430 | 0.1984 | 0.0453 | -0.0112 | 0.1516 | -0.1027 |  |  |  |  |  |  |
|  | **p (no SAD)** | p=0.734 | p=0.487 | p=0.722 | p=0.097 | p=0.707 | p=0.926 | p=0.207 | p=0.394 |  |  |  |  |  |  |
|  | **r (SAD)** | -0.2204 | 0.1067 | 0.3027 | -0.0106 | -0.0793 | -0.2855 | 0.0863 | 0.0665 |  |  |  |  |  |  |
|  | **p (SAD)** | p=0.128 | p=0.466 | p=0.034 | p=0.942 | p=0.588 | p=0.047 | p=0.556 | p=0.650 |  |  |  |  |  |  |
|  | **z** | 0.9589 | -1.0010 | -1.4115 | 1.1088 | 0.6537 | 1.4796 | 0.3471 | -0.8887 |  |  |  |  |  |  |
|  | **p** | p=0.338 | p=0.317 | p=0.158 | p=0.268 | p=0.513 | p=0.139 | p=0.729 | p=0.374 |  |  |  |  |  |  |
| **emotional neglect** | **r (no SAD)** | 0.3113 | -0.3960 | -0.3245 | 0.3958 | -0.4942 | 0.2369 | -0.3365 | -0.3010 |  |  |  |  |  |  |
|  | **p (no SAD)** | p=0.008 | p<0.001 | p=0.006 | p<0.001 | p<0.001 | p=0.047 | p=0.004 | p=0.011 |  |  |  |  |  |  |
|  | **r (SAD)** | -0.4196 | 0.3465 | 0.2969 | -0.1919 | 0.2387 | -0.3754 | 0.3396 | 0.3309 |  |  |  |  |  |  |
|  | **p (SAD)** | p=0.003 | p=0.015 | p=0.038 | p=0.186 | p=0.099 | p=0.008 | p=0.017 | p=0.020 |  |  |  |  |  |  |
|  | **z** | 4.0292 | -4.0877 | -3.3670 | 3.2108 | -4.1120 | 3.3324 | -3.6865 | -3.4282 |  |  |  |  |  |  |
|  | **p** | **p<0.001*** | **p<0.001*** | p<0.001 | p=0.001 | **p<0.001*** | **p<0.001*** | **p<0.001*** | p<0.001 |  |  |  |  |  |  |
| **physical neglect** | **r (no SAD)** | 0.3126 | -0.3661 | -0.2829 | 0.4067 | -0.3439 | 0.2337 | -0.1700 | -0.2732 |  |  |  |  |  |  |
|  | **p (no SAD)** | p=0.008 | p=0.002 | p=0.017 | p<0.001 | p=0.003 | p=0.050 | p=0.156 | p=0.021 |  |  |  |  |  |  |
|  | **r (SAD)** | -0.2079 | 0.2403 | 0.2777 | -0.1048 | 0.1685 | -0.2441 | 0.3098 | 0.2259 |  |  |  |  |  |  |
|  | **p (SAD)** | p=0.152 | p=0.096 | p=0.053 | p=0.473 | p=0.247 | p=0.091 | p=0.030 | p=0.119 |  |  |  |  |  |  |
|  | **z** | 2.7993 | -3.2948 | -3.0173 | 2.8121 | -2.7691 | 2.5522 | -2.5771 | -2.6724 |  |  |  |  |  |  |
|  | **p** | p=0.005 | p<0.001 | p=0.003 | p=0.005 | p=0.006 | p=0.011 | p=0.021 | p=0.008 |  |  |  |  |  |  |
| **total CTQ score** | **r (no SAD)** | 0.4271 | -0.4330 | -0.3755 | 0.5040 | -0.3969 | 0.3158 | -0.2799 | -0.3561 |  |  |  |  |  |  |
|  | **p (no SAD)** | p<0.001 | p<0.001 | p=0.001 | p<0.001 | p<0.001 | p=0.007 | p=0.018 | p=0.002 |  |  |  |  |  |  |
|  | **r (SAD)** | -0.4029 | 0.3024 | 0.3616 | -0.1691 | 0.1737 | -0.4066 | 0.3288 | 0.2823 |  |  |  |  |  |  |
|  | **p (SAD)** | p=0.004 | p=0.035 | p=0.011 | p=0.245 | p=0.233 | p=0.004 | p=0.021 | p=0.049 |  |  |  |  |  |  |
|  | **z** | 4.6277 | -4.0635 | -4.0519 | 3.7997 | -3.1190 | 3.9732 | -3.2951 | -3.4708 |  |  |  |  |  |  |
|  | **p** | **p<0.001*** | **p<0.001*** | **p<0.001*** | **p<0.001*** | **p<0.001*** | **p<0.001*** | **p<0.001*** | **p<0.001*** |  |  |  |  |  |  |

CAL= left sulcus calcarinus/precuneus; MFG = left middle frontal gyrus; sACC=subgenual anterior cingulate cortex; For abbreviations of anatomical structures, see Fig. 3 and Tables S4-7. * after Holm-Bonferroni correction for multiple testing for 36 (CTQ total score) and 180 (subscales) connections significant for p<0.05.

Supplementary Table S11. Interaction of SAD and ELA: spatial convergence of the resting state connectivity patterns of dlPFC[3] and MFG (MVPA, current study).

| **Anatomical region** | **Peak voxel MNI coordinates** | **Cluster size** | **p (FDR)** |
| --- | --- | --- | --- |
| right postcentral gyrus, right precentral gyrus | 42, -10, 30 | 358 | p<0.001 |
| right precentral gyrus, right paracentral lobule, right postcentral gyrus, right supplementary motor area, left paracentral lobule | 8, -28, 56 | 403 | p<0.001 |
| left lingual gyrus, left calcarine fissure and surrounding cortex | -10, -62, 02 | 206 | p<0.001 |
| left postcentral gyrus, left precentral gyrus | -56, -10, 30 | 199 | p<0.001 |
| right lingual gyrus, right calcarine fissure and surrounding cortex | 6, -76, -04 | 200 | p<0.001 |
| right lingual gyrus, right calcarine fissure and surrounding cortex | 26, -62, 04 | 160 | p=0.002 |
| left postcentral gyrus, left inferior parietal gyrus but supramarginal and angular gyri, left precentral gyrus | -40, -10, 34 | 116 | p=0.006 |
| left cuneus, left superior occipital gyrus | -08, -82, 28 | 127 | p=0.005 |
| left paracentral lobule, left postcentral gyrus, left precentral gyrus | -22, -32, 64 | 190 | p<0.001 |

dlPFC= dorsolateral prefrontal gyrus from Wiegand et al. [3]; MFG = left middle frontal gyrus; FDR= false discovery rate, voxel size 2x2x2mm^3^

**
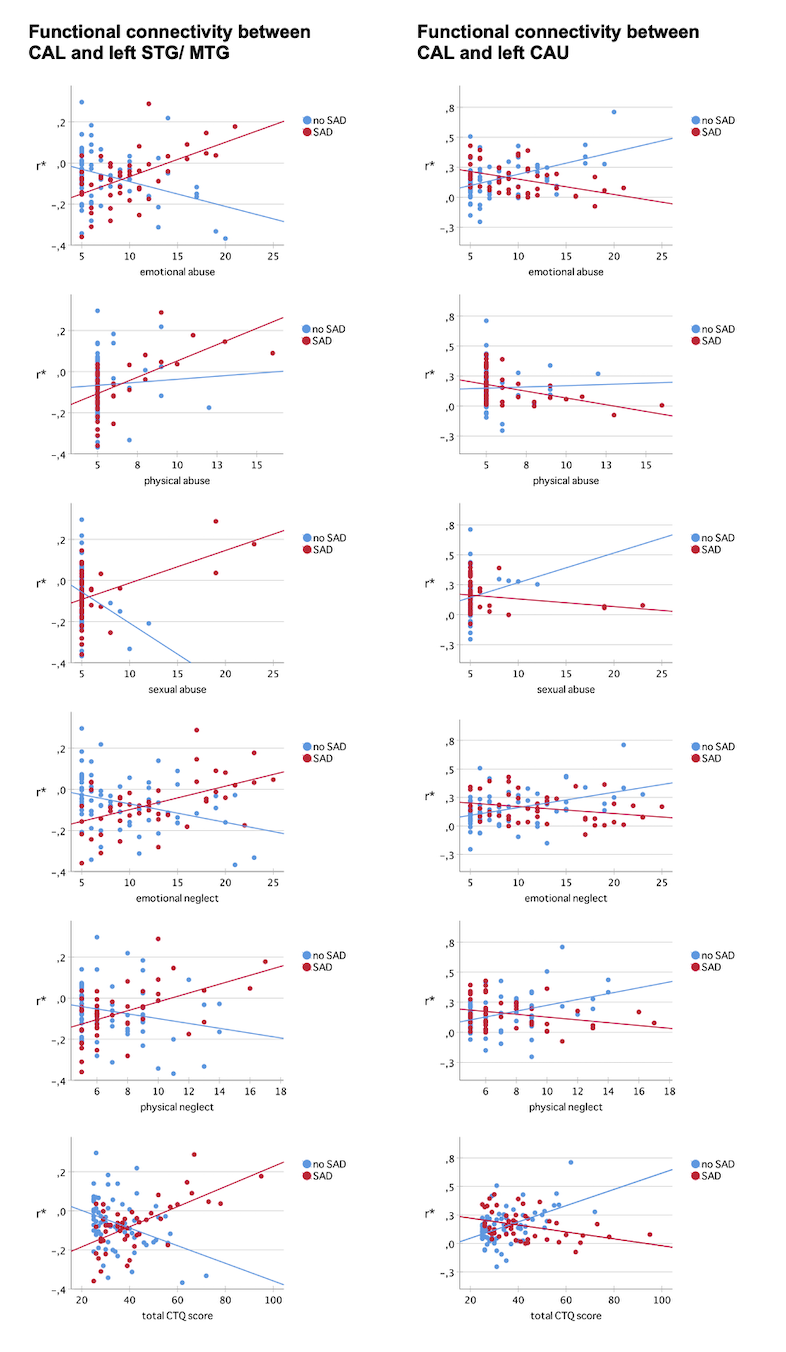
**

Supplementary Figure S2. Association of RSFC with severity of ELA for healthy controls and individuals with SAD. The two linear interaction patterns identified within the functional network are exemplified by the two connections calcarine fissure/ precuneus (CAL) to the left superior temporal gyrus/ middle temporal gyrus (left STG/ MTG); and CAL to the left caudate nucleus (left CAU). Diagrams show Z-transformed bivariate correlation coefficients (r*) of individuals with SAD and controls in relation to the total and subscale scores of the Childhood Trauma Questionnaire (CTQ).


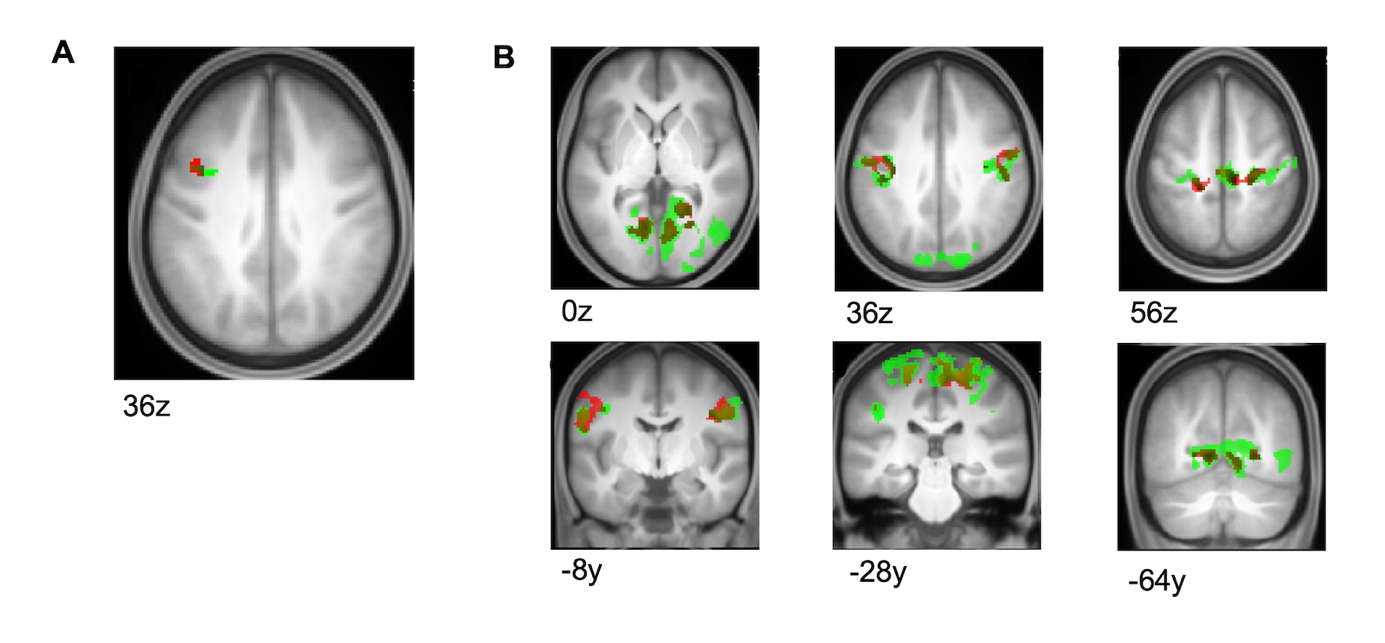


Supplementary Figure S3. Interaction of SAD and ELA: spatial convergence of the resting state connectivity patterns of dlPFC [3] and MFG (MVPA, current study):

A – Spatial congruence of SCA seeds: Red: left dlPFC from Wiegand et al. [3]; green: left middle frontal gyrus (MFG) from the MVPA in the current study.

B – Spatial convergence of SCA results: Red: connectivity pattern of the left dlPFC [3]; green: connectivity pattern of left MFG (MVPA); brown: significant convergences of the connectivity patterns.

**References**

1. Mizzi S, Pedersen M, Lorenzetti V, Heinrichs M, Labuschagne I. Resting-state neuroimaging in social anxiety disorder: a systematic review. *Mol Psychiatry*. 2022;27(1):164-179. doi:10.1038/s41380-021-01154-6

2. Tzourio-Mazoyer N, Landeau B, Papathanassiou D, Crivello F, Etard O, Delcroix N, et al. Automated Anatomical Labeling of Activations in SPM Using a Macroscopic Anatomical Parcellation of the MNI MRI Single-Subject Brain. *Neuroimage*. 2002;15(1):273-289. doi:10.1006/nimg.2001.0978

3. Wiegand A, Munk MHJ, Drohm S, Fallgatter A, MacIsaac J, Kobor M, et al. Neural correlates of attentional control in social anxiety disorder: the impact of early-life adversity and DNA methylation. *Journal of Psychiatry and Neuroscience*. 2021;46(6):E663-E674. doi:10.1503/jpn.210064
