# Supplementary figures and images for: Alterations in cerebral resting state functional connectivity associated with social anxiety disorder and early life adversities
Source: Transl Psychiatry. 2025 Mar 13;15:80. doi: 10.1038/s41398-025-03301-x (PMC11906641; doi:10.1038/s41398-025-03301-x)

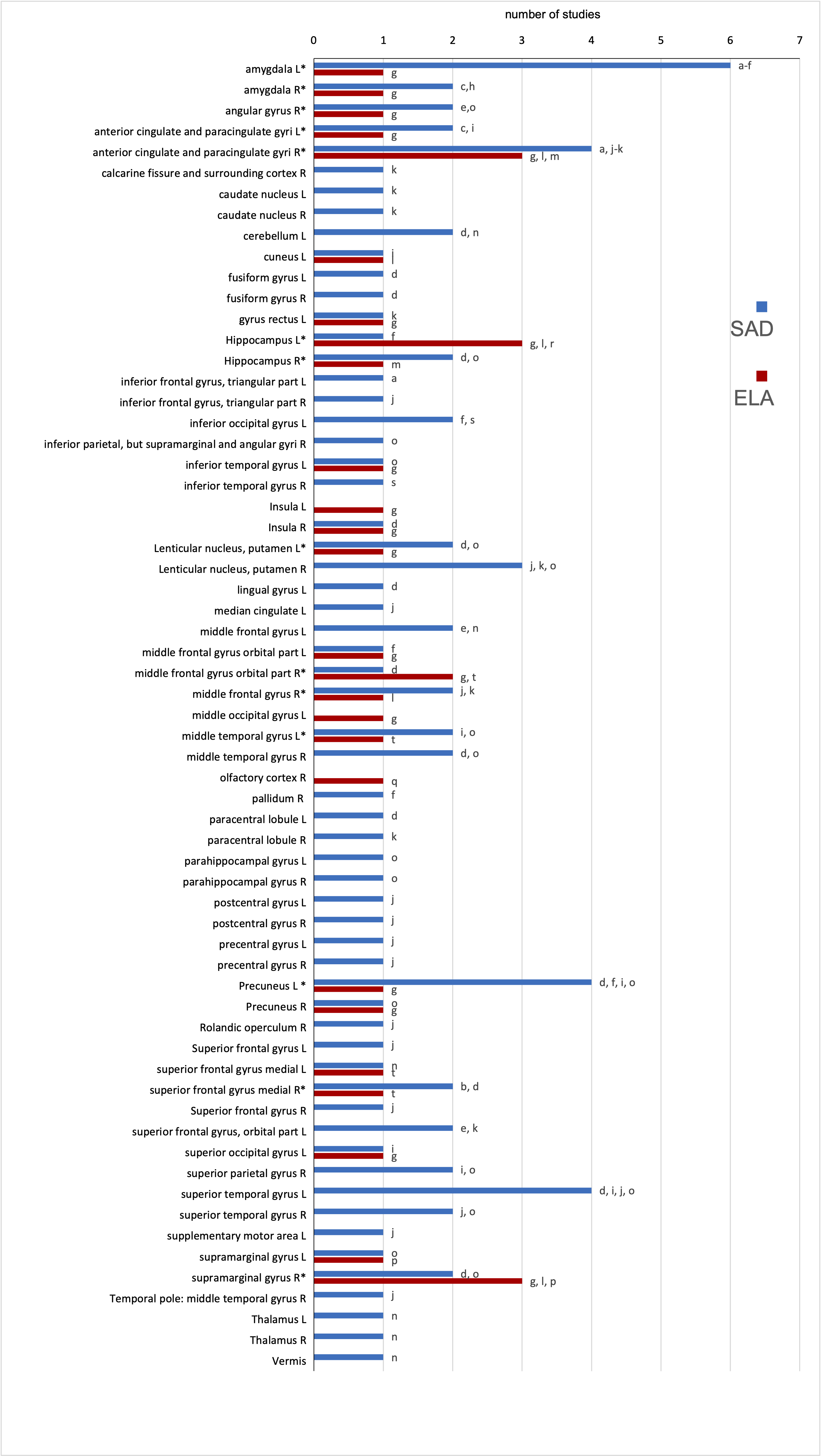

Supplement: Supplementary file 2 — Supplementary Figure 1 [file 41398_2025_3301_MOESM2_ESM.tif]

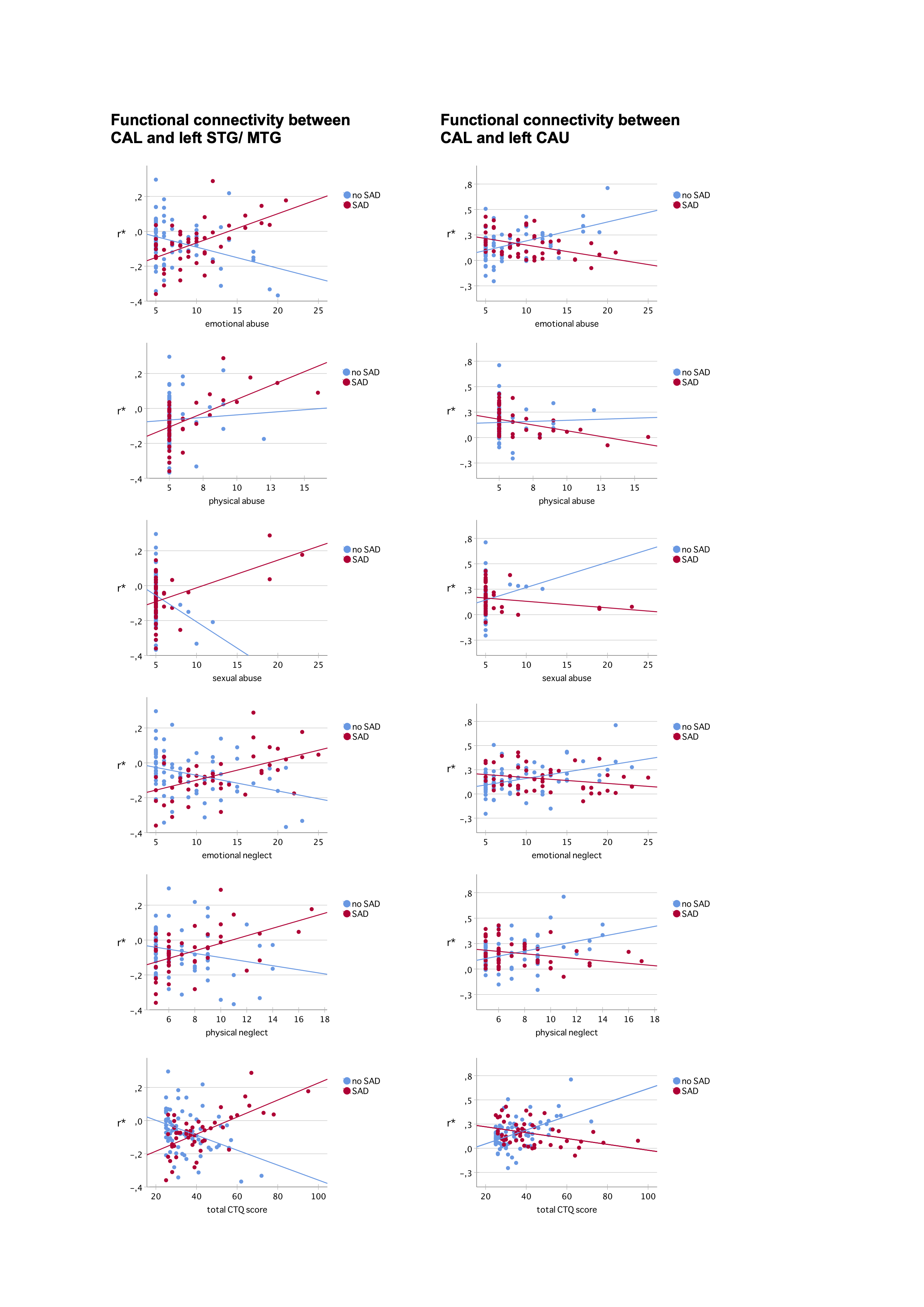

Supplement: Supplementary file 3 — Supplementary Figure 2 [file 41398_2025_3301_MOESM3_ESM.tif]

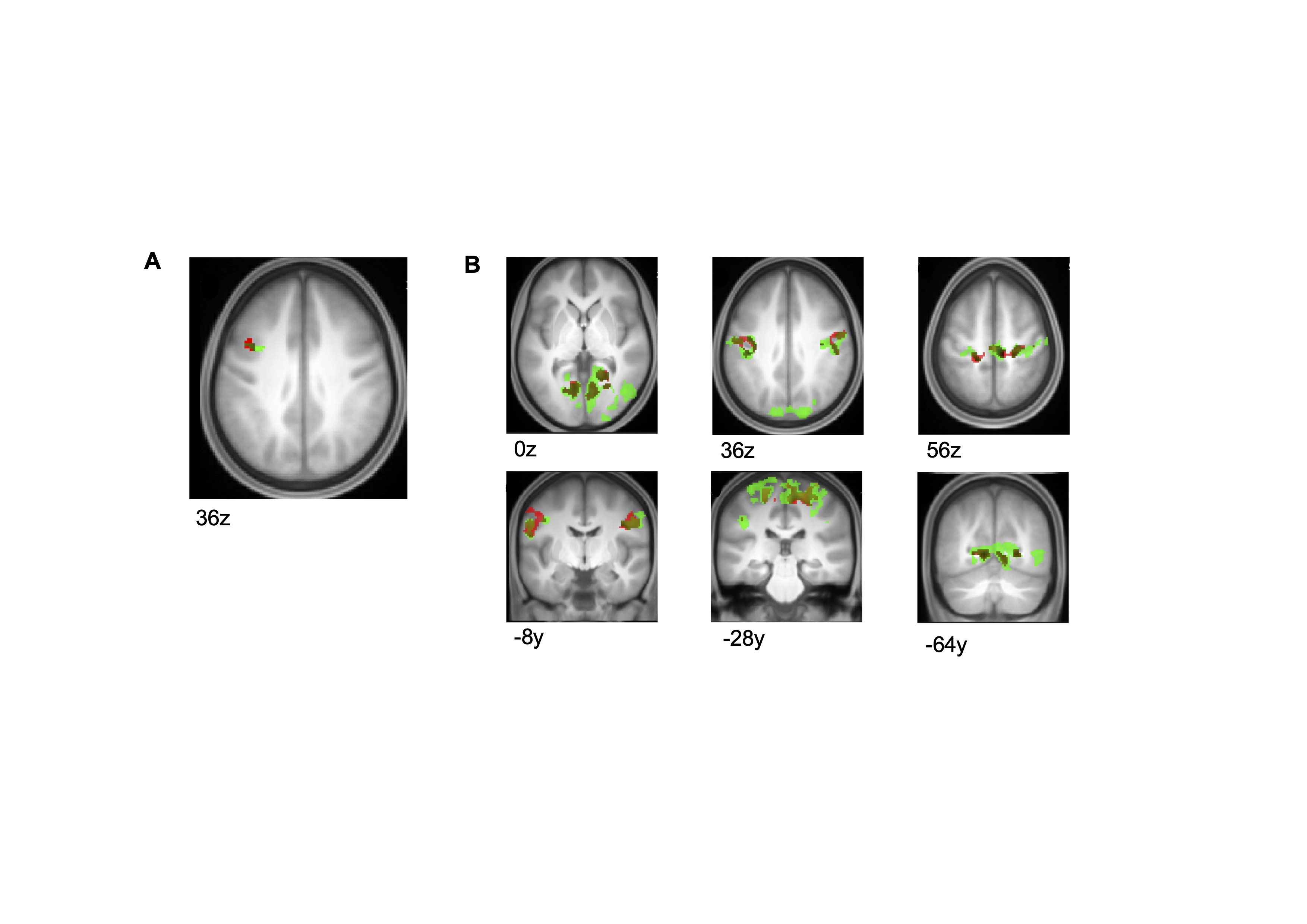

Supplement: Supplementary file 4 — Supplementary Figure 3 [file 41398_2025_3301_MOESM4_ESM.tif]
